# Enabling Photon Upconversion and Precise Control of Donor–Acceptor Interaction through Interfacial Energy Transfer

**DOI:** 10.1002/advs.201700667

**Published:** 2017-12-18

**Authors:** Bo Zhou, Long Yan, Lili Tao, Nan Song, Ming Wu, Ting Wang, Qinyuan Zhang

**Affiliations:** ^1^ State Key Laboratory of Luminescent Materials and Devices and Institute of Optical Communication Materials South China University of Technology Guangzhou 510641 China; ^2^ School of Materials and Energy Guangdong University of Technology Guangzhou 510006 China

**Keywords:** core–shell nanostructures, interfacial energy transfer, mechanistic studies, photon upconversion, spatial control of lanthanides

## Abstract

Upconverting materials have achieved great progress in recent years, however, it remains challenging for the mechanistic research on new upconversion strategy of lanthanides. Here, a novel and efficient strategy to realize photon upconversion from more lanthanides and fine control of lanthanide donor–acceptor interactions through using the interfacial energy transfer (IET) is reported. Unlike conventional energy‐transfer upconversion and recently reported energy‐migration upconversion, the IET approach is capable of enabling upconversions from Er^3+^, Tm^3+^, Ho^3+^, Tb^3+^, Eu^3+^, Dy^3+^ to Sm^3+^ in NaYF_4_‐ and NaYbF_4_‐based core–shell nanostructures simultaneously. Applying the IET in a Nd–Yb coupled sensitizing system can also enable the 808/980 nm dual‐wavelength excited upconversion from a single particle. More importantly, the construction of IET concept allows for a fine control and manipulation of lanthanide donor–acceptor interactions and dynamics at the nanometer‐length scale by establishing a physical model upon an interlayer‐mediated nanostructure. These findings open a door for the fundamental understanding of the luminescence dynamics involving lanthanides at nanoscale, which would further help conceive new scientific concepts and control photon upconversion at a single lanthanide ion level.

## 1 Introduction

Photon upconversion of lanthanides has proved to be an important experimental strategy for realizing the anti‐Stokes type emission, which has been obtained in many kinds of materials greatly promoting their diverse frontier applications in solid‐state lasers, displays, photovoltaics, biological imaging, photodynamic therapy, and nanophotonics.^1^ Benefitting from their unique 4f configuration, the 3+ lanthanides are rich in discrete energy levels, from which the upconverted emissions covering near‐ultraviolet, visible, and near‐infrared spectral region were already obtained in lanthanide‐doped upconversion nanocrystals and bulks.^2^, ^3^ The merits of upconversion nanoparticles including sharp emission bandwidths, large anti‐Stokes shifts, and superior photochemical stability make them to be one class of ideal candidate for the biological nanoprobe.^4^ In addition, their applications in many other frontier fields have further been motivated by the important progresses just recently obtained such as for super‐resolution nanoscopy,^5^ information security and encryption,^6^ and in‐depth description of basic physical phenomena like Brownian motion.^7^

However, to date the design for the most efficient upconversion of nanoparticles remains the energy‐transfer upconversion (ETU), involving a sensitizer‐activator coupled scheme, which was experimentally discovered by Auzel in 1966 for bulk material.^2^ In such a system, the lanthanide dopants are usually incorporated in host matrix simultaneously during the synthetic procedures, making their distributions randomly in the 3D space. This makes their interactions very complex and would unavoidably cause nonradiative decays in particular at heavy doping levels, consequently resulting in a luminescence quenching effect.^2^, ^8^ Moreover, the ETU approach is only efficient for the upconversion of Yb–A (A = Er, Tm, Ho) coupled nanosized systems with infrared irradiation at 980 nm; while for the other upconversion systems or pump schemes, a complicated energy manipulation process and/or structure design have to be adopted.^9^ On the other hand, the precise control of lanthanide interactions at the nanometer‐length scale remains challenging because it is technically impossible for the conventional bulk host materials such as single crystals and glasses.^2^ This impedes the fine manipulating of the upconversion dynamics occurring in lanthanide activators and thereafter the understanding of fundamental energetic processes. The success in experimental synthesis of high quality nanostructured particles has got access into the spatial distribution of lanthanides at nanoscale or even at the sub‐nanometer‐length scale.[[qv: 1a,3]] Therefore, the search for new mechanistic and experimental strategy to realize efficient photon upconversion from more lanthanides and pump schemes would make a substantial contribution to both fundamental research and practical application of upconversion materials.

**Figure 1:**
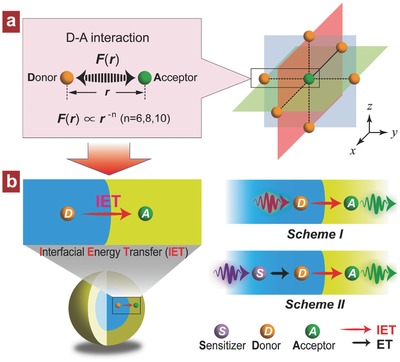
a) Schematic of the interionic interaction between lanthanide energy donor (D) and luminescent acceptor (A) for D–A coupled system. b) Mechanistic illustration of spatially controlling lanthanides in core–shell nanostructure for upconversion through the way of direct interfacial energy transfer (IET) with excitation scheme I or II.

Herein, we describe a novel and efficient mechanistic strategy to generally realize the photon upconversion from more lanthanides through the interfacial energy transfer (IET) in core–shell nanostructures (**Figure** **1**). In our proposal, a suitable lanthanide ion is preselected as the energy donor (D), which together with the luminescent acceptor (A) is incorporated separately in different layers of a core–shell structure, upon which the IET is well established. Under infrared irradiation, the donor in core can be activated in advance by a direct absorption of the excitation energy (Scheme I, Figure 1b) or by a predesigned sensitizer system (Scheme II, Figure 1b), leading to the upconverted emissions from acceptor in shell layer by the IET‐mediated energy‐managing approach. A spatial separation of lanthanide D and A can effectively minimize the deleterious interactions between them in contrast to the conventional D–A codoped scheme (Figure 1a), and it would also be much helpful to enable upconversion from more lanthanides and with more excitation schemes. Moreover, the core–shell‐based design would contribute to the precise manipulation of energy transportation at the sub‐nanometer‐length scale via a finely controllable nanostructure, which is of great importance for an in‐depth understanding of the underlying luminescence physics, particularly at a single ion level.

## 2 Results and Discussion

**Figure 2:**
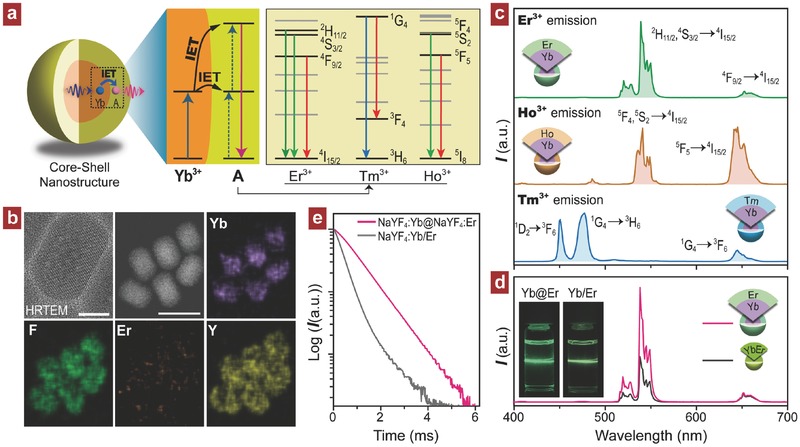
a) Mechanistic illustration of photon upconversion through Yb‐mediated IET for the Yb–A (A = Er, Tm, Ho) coupled core–shell system. b) High resolution TEM image (scale bar, 10 nm) and element mappings of Y, Yb, Er, and F (scale bar, 50 nm) for the as‐synthesized NaYF_4_:Yb (40 mol%)@NaYF_4_:Er (2 mol%) core–shell nanoparticles. c) Upconversion emission spectra obtained from the NaYF_4_:Yb (40 mol%)@NaYF_4_:A (A = Er, 2 mol%; Tm, 1 mol%; Ho, 2 mol%) core–shell samples under 980 nm excitation. d) A comparison of the upconversion emissions from NaYF_4_:Yb (40 mol%)@NaYF_4_:Er (2 mol%) core–shell sample and NaYF_4_:Yb/Er (20/2 mol%) control particles under 980 nm excitation. e) Decay curves of Er^3+^ emissions at 539 nm obtained from (d) samples under a pulsed 980 nm laser excitation.

As a proof of concept, we first investigated the photon upconversion performance from the conventionally studied lanthanide ions including Er^3+^, Tm^3+^, and Ho^3+^ through the IET strategy. In this scheme, Yb^3+^ is adopted as the energy donor because of its capability in absorption of infrared excitation at 980 nm (^2^F_5/2_ ← ^2^F_7/2_ transition).^10^ As shown in **Figure** **2**a, Yb^3+^ and luminescent activator A (A = Er, Tm, Ho) are spatially separated into different layers of the core–shell nanostructure, which were synthesized using a two‐step coprecipitation method (Figure S1a, Supporting Information). Epitaxial growth of a shell layer outside core seeds leads to an increment of resultant nanoparticles in size, which remain the hexagonal phase according to powder X‐ray diffraction (XRD) diffraction profiles (Figure S1b, Supporting Information). Moreover, the spatial separation of Yb^3+^ and Er^3+^ in respective core and shell layers is clearly observed in the element mapping images (Figure 2b). Upon a 980 nm laser irradiation, typical upconversion emission bands of Er^3+^, Tm^3+^, and Ho^3+^ from the NaYF_4_:Yb(40 mol%)@NaYF_4_:A (A = Er, Tm, Ho) core–shell samples were recorded (Figure 2c and Figures S2 and S3 (Supporting Information)). In contrast, without doping of Yb^3+^ in the core area, almost no upconversion was recorded for the control NaYF_4_@NaYF_4_:A core–shell nanoparticles because of the much low absorption at 980 nm (for Er^3+^) or the absence of energy levels matching with the 980 nm laser photon energy (for Tm^3+^ and Ho^3+^) as shown in Figure S4 (Supporting Information). These results have clearly evidenced the validity of Yb^3+^‐mediated IET strategy for realizing the upconverting emissions from conventional upconversion lanthanides.

The NaYF_4_:Yb/A (A = Er, Tm, Ho) upconversion nanoparticles were further prepared as control experiment and their upconversion emission spectra were measured. The emission of Er^3+^ from the NaYF_4_:Yb@NaYF_4_:Er core–shell sample is obviously enhanced than that from the NaYF_4_:Yb/Er nanoparticles (Figure 2d). This can be ascribed to the spatial separation of Yb^3+^ and Er^3+^ in the core–shell structure, which can get the complex randomly spatial interactions to be confined in the interfacial area and subsequently reduce the deleterious D–A interaction (such as back energy transfer occurred at heavy doping levels, see Figure S5 in the Supporting Information).[[qv: 10e,11]] This was further supported by the decay curves that show a much prolonged lifetime in contrast to the NaYF_4_:Yb/Er nanoparticles (Figure 2e). The quantum yield was also measured for the two samples and it is obtained to be 0.15% for NaYF_4_:Yb@NaYF_4_:Er, slightly higher than that of NaYF_4_:Yb/Er (0.13%), may be owing to the increase of optimized Yb^3+^ concentration (from 20 to 40 mol%). Note that the upconversion emissions of Ho^3+^ and Tm^3+^ by the IET process are weaker than that from NaYF_4_:Yb/Ho and NaYF_4_:Yb/Tm samples (Figure S6, Supporting Information). This might be owing to the small D–A interacting range as well as large energy mismatching which usually results in weaker upconversion compared to the NaYF_4_:Yb/Er counterpart nanoparticles under identical measurement condition (Figure S7, Supporting Information). The much slower declining tendency of upconversions of Tm^3+^ and Ho^3+^ than that of Er^3+^ from the samples with heavy doping of Yb^3+^ may also be a reason (Figure S5, Supporting Information). The total upconversion dynamic processes involving such a Yb^3+^‐mediated IET approach is schematically summarized in Figure S8 (Supporting Information).

**Figure 3:**
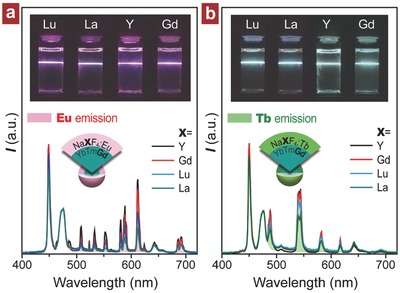
Upconversion emission spectra obtained from the NaYbF_4_:Tm/Gd (1/50 mol%)@NaXF_4_:A (X = Lu, La, Y, Gd) core–shell nanoparticles for a) A = Eu (5 mol%) and b) A = Tb (5 mol%) under 980 nm excitation. Insets show the emission photos taken at the 808/980 nm dual wavelength excitations. Note that the spectral data are normalized to Tm^3+^ emission at 477 nm.

**Figure 4:**
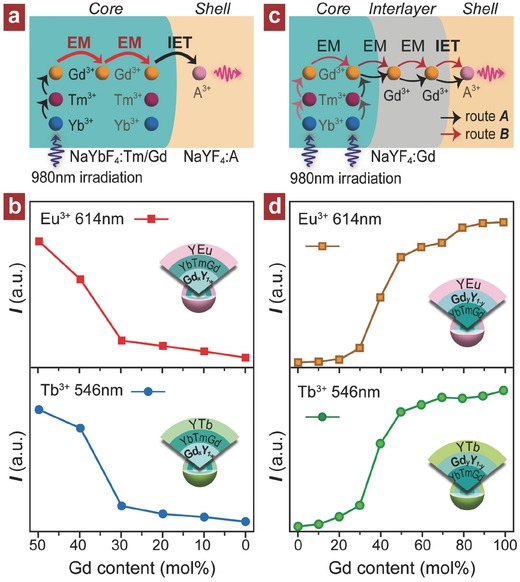
a) Schematic of enabling photon upconversion through energy migration by using the Gd^3+^ donors that locate far away from the core/shell interfacial area. b) Intensity dependence of upconversion emissions on Gd^3+^ content in the inner‐core for the NaYbF_4_:Tm/Gd*_x_*/Y_0.5−_ *_x_*(*x* = 0–0.5)@NaYbF_4_:Tm/Gd (1/50 mol%)@NaYF_4_:A (A = Eu, Tb; 5 mol%) core–shell–shell nanoparticles. c) Illustration of the proposed trilayer structure for examining the energy migration by tuning Gd^3+^ concentration in the interlayer. d) Upconversion emission intensity as a function of Gd^3+^ content in the interlayer for NaYbF_4_:Tm/Gd (1/50 mol%)@NaYF_4_:Gd (0–100 mol%)@NaYF_4_:A (A = Eu, Tb; 5 mol%) core–shell–shell nanoparticles. Note that all upconversion emission spectra were measured at 980 nm irradiation and normalized to Tm^3+^ emission at 477 nm.

Apart from Er^3+^, Tm^3+^, and Ho^3+^, the IET‐induced upconversion from the lanthanide ions without physically existed intermediate states (such as Tb^3+^ and Eu^3+^) can be obtained by using Gd^3+^ as an energy donor in the NaYbF_4_:Tm/Gd@NaYF_4_:A (A = Eu, Tb, Dy, Sm) nanostructure.[[qv: 9f]] We further find that such upconversion holds an independent characteristic on the composition of shell layer matrix as evident by the observation of emissions from the NaYbF_4_:Tm/Gd@NaXF_4_:A (X = Lu, La, Y, Gd) core–shell nanoparticles (**Figure** **3** and Figure S9 (Supporting Information)). This finding proved that the Gd^3+^‐mediated IET is a much more general and efficient approach for upconversion of lanthanides in contrast to the reported pathway of energy migration‐mediated upconversion.[[qv: 9a]] However, considering the importance of energy migratory Gd^3+^ sublattice, it is still worth for us to carry out a comprehensive investigation on the role of Gd^3+^. As illustrated in inset of **Figure** **4**a, we first designed and synthesized a trilayer nanostructure by using a dual‐layer NaYbF_4_:Tm/Gd core where the Gd^3+^ concentration is only variable in the inner core (i.e., NaYbF_4_:Tm/Gd*_x_*/Y_0.5−_ *_x_*@NaYbF_4_:Tm/Gd@NaYF_4_:A). A rapid decline of emission intensity in these samples is clearly observed with reducing concentration of Gd^3+^ in the inner core area (Figure 4b and Figure S10 (Supporting Information)). Thus, it is confirmed that the Gd^3+^ ions residing far away from the interfacial area indeed have a contribution to the photon upconversion through a way of energy migration over the Gd‐sublattice. This result also suggests that the energy migration process is primarily confined in the core other than the shell layer for the NaGdF_4_:Yb/Tm@NaGdF_4_:A upconversion nanoparticles. To investigate the effect of Gd^3+^–Gd^3+^ distance on bridging the energy gap through energy migration, we further prepared a set of control trilayer samples with Gd^3+^ concentration in the interlayer lifted from 0 to 100 mol% (Figure 4c). The upconversion emissions of both Tb^3+^ and Eu^3+^ give a monotonic increase in luminescence intensity with the increase of Gd^3+^ content in interlayer, and then show a saturation tendency when it reaches 50 mol% (Figure 4d and Figure S11 (Supporting Information)). This means that 50 mol% Gd^3+^ is an acceptable level for facilitating the energy migration efficiently, being consistent with the NaYbF_4_:Tm/Gd (50 mol%) used. It should be noted that the Gd^3+^ ions act as energy donor at the core–shell interfacial area would allow the photon upconversion achievable at more excitation schemes.

**Figure 5:**
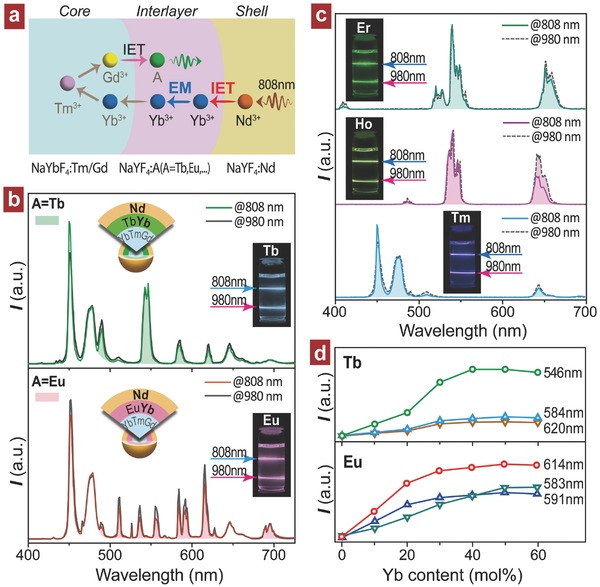
a) Mechanistic illustration of energy transportation pathways in a Nd–Yb coupled trilayer nanostructure for upconversion in the Gd‐mediated system with 808 nm excitation. b) Upconversion emission spectra obtained from NaYbF_4_:Tm/Gd (1/50 mol%)@NaYF_4_:Yb/A (A = Tb, Eu; 40/5 mol%)@NaYF_4_:Nd (40 mol%) core–shell–shell nanoparticles at 808 nm irradiation. Note that the emission spectra measured upon a 980 nm laser were also plotted for comparison and insets show the emission photos taken at the 808/980 nm dual wavelength excitations. c) Upconversion emission spectra obtained from NaYF_4_:Yb/A (A = Er, Tm, Ho; 20/2, 30/1, 20/2 mol%)@NaYF_4_:Nd (40 mol%) core–shell nanoparticles at 808 and 980 nm dual‐wavelength irradiation. d) Upconversion emission intensity of Tb^3+^ and Eu^3+^ as a function of Yb^3+^ concentration in the interlayer (0–60 mol%) for NaYbF_4_:Tm/Gd (1/50 mol%)@NaYF_4_:Yb/A (A = Tb, Eu; 5 mol%)@NaYF_4_:Nd (40 mol%) trilayer samples under 808 nm excitation. Note that the data are normalized to Tm^3+^ emission at 477 nm.

Next, we examined the feasibility of realizing photon upconversion through an IET approach with 808 nm excitation, which shows a greater advantage in diverse biological applications due to the much lower absorption of OH^−^ in comparison to the 980 nm wavelength.^12^ In this case, the Yb^3+^‐sensitized upconversion systems are useless due to the lack of spectral response to the excitation at around 800 nm (Figure S12, Supporting Information). Alternatively, Nd^3+^ shows to be a possible candidate as sensitizer due to its high absorption capability at 808 nm (^4^F_5/2_ ← ^4^I_9/2_ transition) as well as efficient energy transfer from Nd^3+^ to Yb^3+^,^13^ upon which upconversion emissions from Er^3+^, Tm^3+^, and Ho^3+^ were obtained.[[qv: 10e,14]] However, it remains challenging for the photon upconversion from activators such as Tb^3+^ and Eu^3+^ at 808 nm irradiation. Here, we propose an IET‐mediated experimental approach to realize efficient photon upconversion from these lanthanides by using a Nd–Yb coupled core–shell–shell structure (**Figure** **5**a and Figure S13 (Supporting Information)). In detail, an outermost NaYF_4_:Nd sensitizing layer together with a Yb^3+^‐combined interlayer (NaYF_4_:A/Yb interlayer) was adopted to assist the absorption and thereafter the transportation of 808 nm excitation energy. As expected, typical upconversion emissions of Tb^3+^ and Eu^3+^ from these trilayer samples were recorded under 808 nm irradiation, confirming the effectiveness of the trilayer structure proposed (Figure 5b and Figures S14 and S15 (Supporting Information)). In a control, there is almost no emission observed for the samples with both Yb^3+^ and Nd^3+^ (or only using Nd^3+^) in one layer as a result of the strong deleterious interaction between Nd^3+^ and activator A^3+^ (Figure S16, Supporting Information).

Intriguingly, such a trilayer structure endows the samples dual‐wavelength excitable at 980 nm besides the 808 nm owing to the presence of Yb^3+^ in the interlayer matrix (Figure 5b and Figure S17a (Supporting Information)), and more importantly, they present almost identical emission colors (Figure 5b insets) and radiative decays (Figure S17b, Supporting Information). The details of energy transportation involving this dual‐wavelength excitable system were schematically illustrated in Figure S18 (Supporting Information). In addition, the 980/808 nm dual‐wavelength excited upconversion was also observed for Er^3+^, Tm^3+^, and Ho^3+^ by constructing a NaYF_4_:Yb/A (A = Er, Tm, Ho)@NaYF_4_:Nd core–shell nanostructure (Figure 5c and Figures S19–S22 (Supporting Information)). Thus, by using IET‐mediated core–shell strategy, a nanostructure design was established for efficient photon upconversion at 980/808 nm dual‐wavelength excitation from a single particle.

Another important phenomenon observed from Figure 5b is the energy migration effect among Yb^3+^ ions that plays an essential role in facilitating the transportation of 808 nm excitation energy from the outermost layer to the core. Experimental construction of trilayer nanostructure permits a possibility for investigating the detail of Yb^3+^‐mediated energy migration. Hence, we synthesized NaYbF_4_:Tm/Gd@NaYF_4_:A/Yb@NaYF_4_:Nd trilayer samples in which the Yb^3+^ concentration in interlayer is variable. Their spectral results (Figure 5d) reveal an increasing luminescence intensity change with Yb^3+^ content, and a saturation effect occurring at around 30 mol% of Yb^3+^, corresponding to an average Yb^3+^–Yb^3+^ separation of 0.77 nm. Unlikely Gd^3+^, much more Yb^3+^ ions preside in the energy migratory route does not contribute to a further enhancement of upconversion. To shed more light on the energy migratory role of Yb^3+^, we studied the emission property of NaYbF_4_:Tm/Gd@NaYF_4_:A/Yb core–shell samples with different Yb^3+^ concentrations in the shell layer. The spectral results show a rapid decrease other than further increase in emission intensity at higher Yb^3+^ dopant concentration (>20 mol%) under 980 nm excitation (Figure S23, Supporting Information), being different from the result exhibited in Figure 5d where a third NaYF_4_:Nd layer grew on these core–shell particles. This discovery confirmed that the presence of Yb^3+^ in shell sublattice can conduct the energy to particle surface, consequently resulting in a severe luminescence quenching. Thus, a coating of outermost NaYF_4_:Nd layer on these core–shell nanoparticles prohibits the energy loss arising from the Yb^3+^‐mediated energy migration, which further imparts the photon upconversion with a dual‐wavelength excitation character. The result is in agreement with the fact that only limited Yb^3+^ content is incorporated in the protective shell layer for enhancing upconversion in core–shell systems (e.g., NaGdF_4_:Yb/Er@NaGdF_4_:Yb nanoparticles).^15^

**Figure 6:**
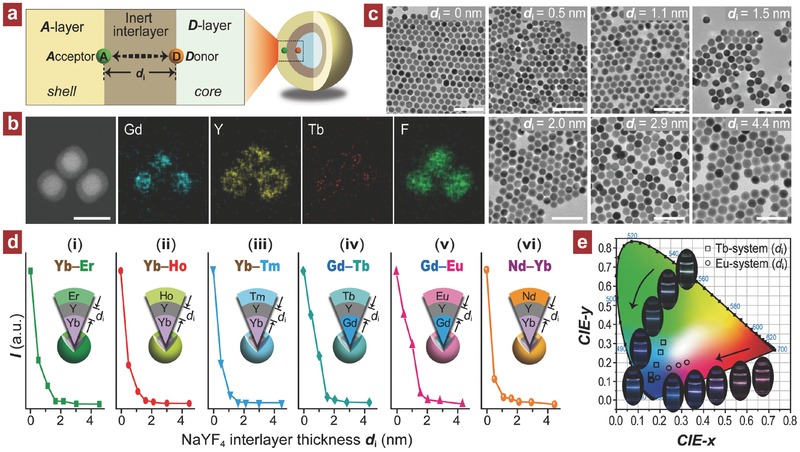
a) Schematic of controlling donor–acceptor energy transfer in nanostructure by precisely mediating the inactive interlayer thickness in the proposed core–shell–shell trilayer structure. b) Element mapping images of Gd, Y, Tb, and F for the as‐prepared trilayer NaYbF_4_:Tm/Gd@NaYF_4_@NaYF_4_:Tb nanoparticles. Scale bar, 50 nm. c) TEM images of NaYbF_4_:Tm/Gd core seeds coating with different NaYF_4_ interlayer thicknesses (0–4.4 nm). Scale bars, 100 nm. d) Upconversion emission intensity as a function of interlayer thickness for (i–iii) NaYF_4_:Yb (40 mol%)@NaYF_4_@NaYF_4_:A (A = Er, 2 mol%; Ho, 2 mol%; Tm, 0.5 mol%) and (iv, v) NaYbF_4_:Tm/Gd (1/50 mol%)@NaYF_4_@NaYF_4_:A (A = Eu, Tb; 5 mol%) core–shell–shell nanocrystals under 980 nm excitation, and (vi) NaYF_4_:Yb/Er (20/2 mol%)@NaYF_4_@NaYF_4_:Nd (40 mol%) core–shell–shell nanocrystals under 808 nm excitation. Note that the emissions used are from (i) Er^3+^ at 539 nm, (ii) Tm^3+^ at 477 nm, (iii) Ho^3+^ at 541 nm, (iv) Eu**^3+^** at 614 nm, (v) Tb^3^ **^+^** at 545 nm, and (vi) Er^3+^ at 539 nm, respectively. e) CIE(*x*,*y*) chromaticity diagram of emission colors and corresponding emission photos taken at 980 nm irradiation for the samples with the presence and further increase of interlayer thickness (arrow directions).

Furthermore, the IET‐mediated multilayer nanostructure is capable of providing a physical platform for in‐depth studying of interionic lanthanide interactions at nanoscale. This could be an overwhelming advantage for the insight into the detail of energetic processes involving lanthanides because in the conventional bulk materials, it is technically impossible to control the distribution of lanthanide dopant at nanoscale. In this regard, we propose a core–shell–shell nanoarchitecture to investigate the lanthanide D–A energy transfer at the nanometer‐length scale. As illustrated in **Figure** **6**a, an optically inactive NaYF_4_ interlayer is adopted to spatially separate the donor and acceptor. Through a precise control of the interlayer thickness, the D–A energy transfer can be monitored by recording the emissions from the outermost A‐coupled shell layer. A set of control samples with interlayer thickness variable were synthesized (e.g., Figure 6b,c) using a modified three‐step coprecipitation method (Supporting Information). For the Yb–A (A = Er, Ho, Tm) interactive system, NaYF_4_:Yb@NaYF_4_@NaYF_4_:A trilayer particles were designed and their upconversion emissions show a rapid decline with increasing interlayer thickness from 0 to 4.5 nm (Figure 6d(i–iii) and Figure S24 (Supporting Information)). When the thickness reaches about 1.6 nm, the luminescence intensity drops to a level lower than one order of magnitude, indicating that the effective D–A separation for upconversion of Yb–A system is limited in a range less than 1.6 nm. The Gd–A (A = Tb, Eu) system exhibits a similar spectral result as observed from the NaYbF_4_:Tm/Gd@NaYF_4_@NaYF_4_:A trilayer samples and the effective D–A separation is found at around 2.0 nm (Figure 6d(iv, v) and Figure S25 (Supporting Information)).

Considering the critical role of migratory Yb^3+^ sublattice for the 808 nm pumped photon upconversion, we further examined the energy transfer from Nd^3+^ to Yb^3+^ by building a NaYF_4_:Yb/Er@NaYF_4_@NaYF_4_:Nd trilayer nanostructure. The dependence of Nd^3+^‐to‐Yb^3+^ energy transfer on Nd^3+^–Yb^3+^ separation can be detected by observing upconversion emission from the Yb/Er coupled core area (Figure S26a, Supporting Information). At 808 nm irradiation, the upconverted emission of Er^3+^ is obtained that also presents a rapid decline with increasing the interlayer thickness (Figure 6d(vi) and Figure S26b (Supporting Information)), suggesting an effective Nd^3+^–Yb^3+^ separation limit of 2.1 nm. As an added benefit, the trilayer nanostructure presents a new way to finely tune the emission color of Gd‐mediated upconversion system by modulating the NaYF_4_ interlayer thickness in addition to the tuning of dopant concentration or pump power (Figure 6e).[[qv: 9a]] These results have experimentally evidenced that typical lanthanide D–A interaction distance lies in a short range less than 1.6–2.1 nm for effectively facilitating the energy transfer mediated upconversion.

## 3 Conclusion

In conclusion, we have experimentally demonstrated that interfacial energy transfer is an efficient and more general strategy for achieving the photon upconversion from a series of lanthanides. By constructing an interlayer‐thickness controllable trilayer nanostructure, we further developed a physical model for quantitatively examining the interactions involving lanthanide donor–acceptor pairs (Yb–Er/Tm/Ho, Gd–Eu/Tb, and Nd–Yb) at nanometer levels, and their separation for efficiently facilitating the energy transfer was precisely determined to be confined in a range less than 1.6–2.1 nm. These findings present an in‐depth insight into the mechanistic understanding of upconversion luminescence physics involving lanthanides at the nanometer‐length scale. More significantly, they may help to construct new scientific concepts and ingenious experimental designs for manipulating and controlling photon upconversion at a single lanthanide ion level in the near future.

## 4 Experimental Section

*Materials*: The materials including yttrium(III) acetate hydrate (99.9%), gadolinium(III) acetate hydrate (99.9%), lutetium(III) acetate hydrate (99.9%), lanthanum(III) acetate hydrate (99.9%), ytterbium(III) acetate hydrate (99.99%), neodymium(III) acetate hydrate (99.9%), erbium(III) acetate hydrate (99.9%), holmium(III) acetate hydrate (99.9%), thulium(III) acetate hydrate (99.9%), europium(III) acetate hydrate (99.9%), terbium(III) acetate hydrate (99.9%), dysprosium(III) acetate hydrate (99.9%), samarium(III) acetate hydrate (99.9%), oleic acid (90%), 1‐octadecene (90%), sodium hydroxide (NaOH; >98%), and ammonium fluoride (NH_4_F; >98%) were all purchased from Sigma‐Aldrich, and used as received unless otherwise noted.

*Sample Synthesis*: The core nanoparticles were synthesized using a coprecipitation chemical method, and the core–shell nanoparticles were prepared by a two‐step coprecipitation method through using the presynthesized core nanoparticles as seeds for shell layer growth. The core–shell–shell trilayer nanoparticles were prepared by a similar method to the synthesis of core–shell nanoparticles but using the presynthesized core–shell nanoparticles as seeds for the outermost shell layer growth, and the interlayer thickness‐mediated trilayer nanoparticles were prepared through a fine tuning of the interlayer precursor content. The detailed experimental procedures for synthesis of these nanoparticles samples are provided in the Supporting Information.

*Characterization*: The upconversion luminescence spectra were recorded by a Jobin‐Yvon Triax 320 spectrofluorometer equipped with a 980 nm and an 808 nm laser diodes. The decay curves were measured using the same spectrofluorometer through the use of 980 and 808 nm pulsed laser as excitation sources. Low‐ and high‐resolution transmission electron microscopy (TEM) measurements together with energy‐dispersive X‐ray spectroscopy was carried out on a JEM 2100F TEM (200 kV). The quantum yield of upconversion emission was measured using an integrating sphere method following the procedures described in a recent literature,^16^ and the procedure detail is described in the Supporting Information. XRD data were recorded on a Philips Model PW1830 X‐ray powder diffractometer with Cu Kα radiation (λ = 1.5406 Å). The upconversion emission photographs were taken by a digital camera.

## Conflict of Interest

The authors declare no conflict of interest.

## Supporting information

Supplementary

## References

[1] a) B. Zhou , B. Shi , D. Jin , X. Liu , Nat. Nanotechnol. 2015, 10, 924; 26530022 10.1038/nnano.2015.251

[2] F. Auzel , Chem. Rev. 2004, 104, 139. 14719973 10.1021/cr020357g

[3] a) M. Haase , H. Schäfer , Angew. Chem., Int. Ed. 2011, 50, 5808; 10.1002/anie.201005159 21626614

[4] a) J. C. G. Bunzli , Chem. Rev. 2010, 110, 2729; 20151630 10.1021/cr900362e

[5] Y. Liu , Y. Lu , X. Yang , X. Zheng , S. Wen , F. Wang , X. Vidal , J. Zhao , D. Liu , Z. Zhou , C. Ma , J. Zhou , J. A. Piper , P. Xi , D. Jin , Nature 2017, 543, 229. 28225761 10.1038/nature21366

[6] a) J. Lee , B. Yoo , H. Lee , G. D. Cha , H.‐S. Lee , Y. Cho , S. Y. Kim , H. Seo , W. Lee , D. Son , M. Kang , H. M. Kim , Y. I. Park , T. Hyeon , D.‐H. Kim , Adv. Mater. 2017, 29, 1603169; 10.1002/adma.201603169 27748544

[7] C. D. S. Brites , X. Xie , M. L. Debasu , X. Qin , R. Chen , W. Huang , J. Rocha , X. Liu , L. D. Carlos , Nat. Nanotechnol. 2016, 11, 851. 27376242 10.1038/nnano.2016.111

[8] a) S. Hinojosa , M. A. Meneses‐Nava , O. Barbosa‐Garcia , L. A. Diaz‐Torres , M. A. Santoyo , J. F. Mosino , J. Lumin. 2003, 102–103, 694;

[9] a) F. Wang , R. Deng , J. Wang , H. Wang , Y. Han , H. Zhu , X. Chen , X. Liu , Nat. Mater. 2011, 10, 968; 22019945 10.1038/nmat3149

[10] a) E. Snitzer , R. Woodcock , Appl. Phys. Lett. 1965, 6, 45;

[11] a) G. Chen , H. Liu , H. Liang , G. Somesfalean , Z. Zhang , J. Phys. Chem. C 2008, 112, 12030;

[12] a) R. Weissleder , Nat. Biotechnol. 2001, 19, 316; 11283581 10.1038/86684

[13] a) M. J. Weber , Phys. Rev. B 1971, 4, 3153;

[14] a) Y.‐F. Wang , G.‐Y. Liu , L.‐D. Sun , J.‐W. Xiao , J.‐C. Zhou , C.‐H. Yan , ACS Nano 2013, 7, 7200; 23869772 10.1021/nn402601d

[15] F. Vetrone , R. Naccache , V. Mahalingam , C. G. Morgan , J. A. Capobianco , Adv. Funct. Mater. 2009, 19, 2924.

[16] J.‐C. Boyer , F. C. J. M. van Veggel , Nanoscale 2010, 2, 1417. 20820726 10.1039/c0nr00253d

