# Supplementary material for: Enabling Photon Upconversion and Precise Control of Donor–Acceptor Interaction through Interfacial Energy Transfer
Source: Adv Sci (Weinh). 2017 Dec 18;5(3):1700667. doi: 10.1002/advs.201700667 (PMC5867046; doi:10.1002/advs.201700667)
Supplement: Supplementary file 1 — Supplementary [file ADVS-5-1700667-s001.pdf]

Copyright WILEY-VCH Verlag GmbH & Co. KGaA, 69469 Weinheim, Germany, 2017.

ADVANCED SCIENCE
Open Access

Supporting Information

for *Adv. Sci.,* DOI: 10.1002/advs.201700667

Enabling Photon Upconversion and Precise Control of Donor–Acceptor Interaction through Interfacial Energy Transfer

Bo Zhou, Long Yan, Lili Tao,* Nan Song, Ming Wu, Ting Wang, and Qinyuan Zhang**

Supporting Information

Enabling Photon Upconversion and Precise Control of Donor-Acceptor Interaction through Interfacial Energy Transfer

Bo Zhou,[†,*] Long Yan,[†] Lili Tao,[‡,*] Nan Song,[†] Ming Wu,[†] Ting Wang,[†] and Qinyuan Zhang[†,*]

[†] State Key Laboratory of Luminescent Materials and Devices, and Institute of Optical Communication Materials, South China University of Technology, Guangzhou, 510641, China

[‡] School of Materials and Energy, Guangdong University of Technology, Guangzhou, 510006, China

*Corresponding author

E-mails: (B.Z.) zhoubo@scut.edu.cn

(L.T.) aptaoll@163.com

(Q.Z.) qyzhang@scut.edu.cn

I. Experimental Procedures

1. Synthesis of core nanoparticles

The core nanoparticles were synthesized using a co-precipitation chemical method. In a typical experiment for the synthesis of $NaYF_4$:Yb(40 mol%) core nanoparticle, to a 50-mL flask containing oleic acid (4 mL) and 1-octadecene (6 mL) was added a water solution (2 mL) containing $Y(CH_3CO_2)_3$ and $Yb(CH_3CO_2)_3$ at designed ratios (60:40 mol%) with a total lanthanide amount of 0.4 mmol. The resulting mixture was heated at 150 ^oC for 1 h to form lanthanide oleate complexes and then cooled down to room temperature. Subsequently, a methanol solution containing NaOH (1 mmol) and NH_4F (1.6 mmol) was added and stirred at 50 ^oC for 0.5 h, and then heated at 290 ^oC under an argon flow for 1.5 h before cooling down to room temperature. The resulting core nanoparticles were collected by centrifugation, washed with ethanol, and finally dispersed in cyclohexane.

The synthetic procedure for other core nanoparticles of $NaYF_4$:Yb(20,30,50,60 mol%), $NaYF_4$:Yb/A(A=Er,Tm,Ho; Yb/A=x/y mol%), $NaYbF_4$:Tm/Gd(1/50 mol%), $NaYbF_4$:Tm/Gd/Y(1/50-x/x mol%), $NaYF_4$:Yb/A/Nd(A=Er,Tm,Ho; Yb/A=20/2, 30/0.5, 20/2 mol%; Nd, 40 mol%), $NaYbF_4$:Tm/Gd/Nd(1/50/20 mol%), and $NaYF_4$ was identical to the synthesis of $NaYF_4$:Yb(40 mol%) core nanoparticles except for the use of different lanthanide precursors.

2. Synthesis of core-shell nanoparticles

The core-shell nanoparticles were prepared by a two-step co-precipitation method through using the pre-synthesized core nanoparticles as seeds for shell layer growth. In a typical synthetic procedure of $NaYF_4$:Yb(40 mol%)@$NaYF_4$:Er(2 mol%) core-shell nanoparticle, to a 50-mL flask containing oleic acid (3 mL) and 1-octadecene (7 mL) was added a water solution (2 mL) containing $Y(CH_3CO_2)_3$ and $Er(CH_3CO_2)_3$ at designed ratio (98:2 mol%) with a total lanthanide amount of 0.4 mmol. The mixture was then heated at 150 ^oC for 1 h with magnetic stirring and then cooled down room temperature. Subsequently, the $NaYF_4$:Yb(40 mol%) nanoparticles were added as seeds along with a 6-mL methanol solution of NH_4F (1.6 mmol) and NaOH (1 mmol). The reaction mixture was stirred at 50 ^oC for 0.5 h, and then heated at 290 ^oC under an argon flow for 1.5 h before cooling down to room temperature. The resulting core-shell nanoparticles were collected by centrifugation, washed with ethanol, and dispersed in cyclohexane.

The synthetic procedure for other core-shell nanoparticles including $NaYF_4$:Yb(x mol%)@$NaYF_4$:A(A=Er,Tm,Ho; y mol%), $NaYF_4$:Yb/A@$NaYF_4$, $NaYF_4$:Yb/A (A=Er,Tm,Ho)@$NaYF_4$:Nd, $NaYF_4$@$NaYF_4$:A(A=Er,Tm,Ho), $NaYbF_4$:Tm/Gd(1/50 mol%)@$NaXF_4$:A(X=Y,Gd,Lu,La; A=Tb,Eu,Dy,Sm), $NaYbF_4$:Tm/Gd/Y(1/ 50-x/x mol%)@$NaYbF_4$:Tm/Gd(1/50 mol%), $NaYbF_4$:Tm/Gd(1/50 mol%)@$NaXF_4$:Yb/A(A=Tb,Eu; Yb/A=x/5 mol%), $NaYbF_4$:Tm/Gd(1/50 mol%)@$NaXF_4$:Gd(x mol%), $NaYbF_4$:Tm/Gd(1/50 mol%)@$NaYbF_4$:A/Nd(A=Tb,Eu; Nd, 50 mol%), $NaYF_4$:Yb/A(A=Er,Tm,Ho)@$NaYF_4$:Nd (x mol%), and $NaYF_4$:Yb/A/Nd@$NaYF_4$ was identical to that for $NaYbF_4$:Yb(40 mol%)@$NaYF_4$:Er(2 mol%) core-shell nanoparticles except for the use of different core nanoparticles as seeds and corresponding lanthanide shell precursors.

3. Synthesis of core-shell-shell nanoparticles

The core-shell-shell trilayered nanoparticles were prepared by a three-step co-precipitation method through using the pre-synthesized core-shell nanoparticles as seeds for the outermost shell layer growth. In a typical synthesis of $NaYbF_4$:Tm/Gd(1/50 mol%)@$NaYF_4$:Gd(0-100 mol%)@$NaYF_4$:Tb(5 mol%) tri-layered nanoparticle, to a 50-mL flask containing oleic acid (3 mL) and 1-octadecene (7 mL) was added a water solution (2 mL) containing $Y(CH_3CO_2)_3$ and $Gd(CH_3CO_2)_3$ at designed ratios (e.g., 50:50 mol% with a total lanthanide amount of 0.4 mmol. The mixture was then heated at 150 ^{o}C for 1 h with magnetic stirring and then cooled down room temperature. Subsequently, the pre-synthesized $NaYbF_4$:Tm/Gd(1/50 mol%)@$NaYF_4$:Gd nanoparticles were added as seeds along with a 6-mL methanol solution of NH_4F (1.6 mmol) and NaOH (1 mmol). The reaction mixture was stirred at 50 ^{o}C for 0.5 h, and then heated at 290 ^{o}C under an argon flow for 1.5 h before cooling down to room temperature. The resulting core-shell-shell nanoparticles were collected by centrifugation, washed with ethanol, and dispersed in cyclohexane.

The synthetic procedure for other core-shell nanoparticles including $NaYbF_4$:Tm/Gd(1/50 mol%)@$NaYF_4$:Gd(0-100 mol%)@$NaYF_4$:Eu (5 mol%), $NaYbF_4$:Tm/Gd/Y(1/50-x/x mol%)@$NaYbF_4$:Tm/Gd(1/50 mol%)@$NaYF_4$:A(A = Tb,Eu; 5 mol%), $NaYbF_4$:Tm/Gd(1/50 mol%)@$NaYF_4$:Yb/A(A = Tb,Eu,Dy,Sm; Yb, 0-60 mol%)@$NaYF_4$:Nd(0-60 mol%), and $NaYbF_4$:Tm/Gd(1/50 mol%)@$NaYbF_4$:A/Nd(A = Tb,Eu; Nd, 50 mol%)@$NaYF_4$:Nd(0-60 mol%) core-shell-shell nanoparticles was identical to that for $NaYbF_4$:Tm/Gd(1/50 mol%)@$NaYF_4$:Gd(0-100 mol%)@$NaYF_4$:Tb (5 mol%) core-shell-shell nanoparticles except for the use of different core nanoparticles as seeds and corresponding lanthanide outer shell precursors.

4. Synthesis of interlayer thickness varied core-shell-shell nanoparticles

The interlayer thickness tunable $NaYF_4$:Yb(40 mol%)@$NaYF_4$@$NaYF_4$:Er(2 mol%) tri-layer nanoparticles were synthesized using a similar procedure to that for common core-shell-shell nanoparticles except for a control of the core and intershell lanthanide solution ratio. The intershell lanthanide solution was designed at different ratios based on the thickness growth rate. In a typical experiment, to a 50-mL flask containing oleic acid (4 mL) and 1-octadecene (6 mL) was added a water solution (2 mL) containing γ (γ = 0, 0.04, 0.08, 0.12, 0.16, 0.24, 0.32, 0.40) mmol $Y(CH_3CO_2)_3$. The mixture was heated at 150 ^{o}C for 1 h with magnetic stirring and then cooled down to room temperature. Subsequently, the $NaYF_4$:Yb(40 mol%) core nanoparticles were added as seeds along with a methanol solution of NH_4F (4γ mmol) and NaOH (2.5γ mmol). The reaction mixture was stirred at 50 ^{o}C for 0.5 h, and then heated at 290 ^{o}C under an argon flow for 1.5 h before cooling down to room temperature. The resultantly obtained core-shell nanoparticles were further used as seeds for growing the outermost $NaYF_4$:Er shell layer following the same procedure except for the use of outer shell precursor containing 0.40 mmol $Y(CH_3CO_2)_3$ and $Er(CH_3CO_2)_3$ at a ratio of 98:2 mol%. The resulting thickness controllale core-shell-shell nanoparticles were collected by centrifugation, washed with ethanol and dispersed in cyclohexane.

The intershell layer thickness tunable $NaYbF_4$:Tm/Gd(1/50 mol%)@$NaYF_4$@$NaYF_4$:A (A=Tb,Eu; 5 mol%), and $NaYF_4$:Yb/Er(20/2 mol%)@$NaYF_4$@$NaYF_4$:Nd(40 mol%) tri-layer nanoparticles were synthesized using a similar procedure to that for common core-shell-shell nanoparticles except for a control of the core and outermost shell lanthanide solutions.

5. Measurement of quantum yield

The quantum yield of upconversion emission was measured using an integrating sphere method following the procedures described in ref 1. Typically, a side-polished quartz cuvette containing a cyclohexane solution of the nanoparticles was placed inside the integrating sphere mounted in an Edinburgh spectrometer chamber. A cyclohexane solution without the nanoparticles was also used for reference. All the spectroscopic data were collected using the spectrometer, and they were corrected for the spectral response of detector before being used for calculating the quantum yield (QY) according to the following equation:

$$QY = N_{em} / N_{abs} = L_{samp} / (E_{ref} - E_{samp})$$

where L_{samp} is the upconversion emission intensity of the sample, E_{ref} and E_{samp} are the intensities of the excitation light not absorbed by the sample and the reference, respectively.

Reference

[1] J.-C. Boyer, F. C. J. M. van Veggel, *Nanoscale* **2010**, *2*, 1417.

II. Supplementary Figures

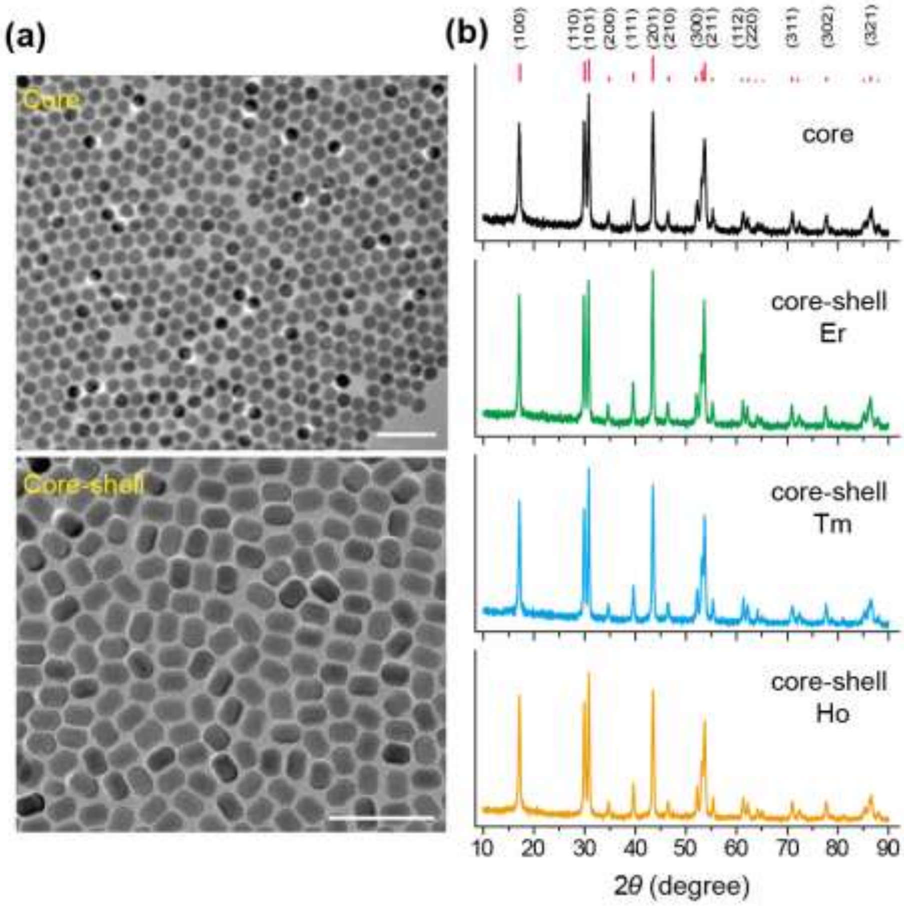

Figure S1. (a) TEM images of as-synthesized $NaYF_4$:Yb(40 mol%) core and $NaYF_4$:Yb(40 mol%)@$NaYF_4$:Er(2 mol%) core-shell nanoparticles showing an increment in size for the core seeds with growing the shell layer. Scale bars, 100 nm. (b) X-ray diffraction (XRD) paterrns of as-synthesized $NaYF_4$:Yb(40 mol%) core and corresponding $NaYF_4$:Yb(40 mol%)@$NaYF_4$:Er(2 mol%), $NaYF_4$:Yb(40 mol%)@$NaYF_4$:Tm(0.5 mol%), and $NaYF_4$:Yb(40 mol%)@$NaYF_4$:Ho(2 mol%) core-shell nanoparticles, showing haxagnoal phase feature for all the nanoparticle samples.

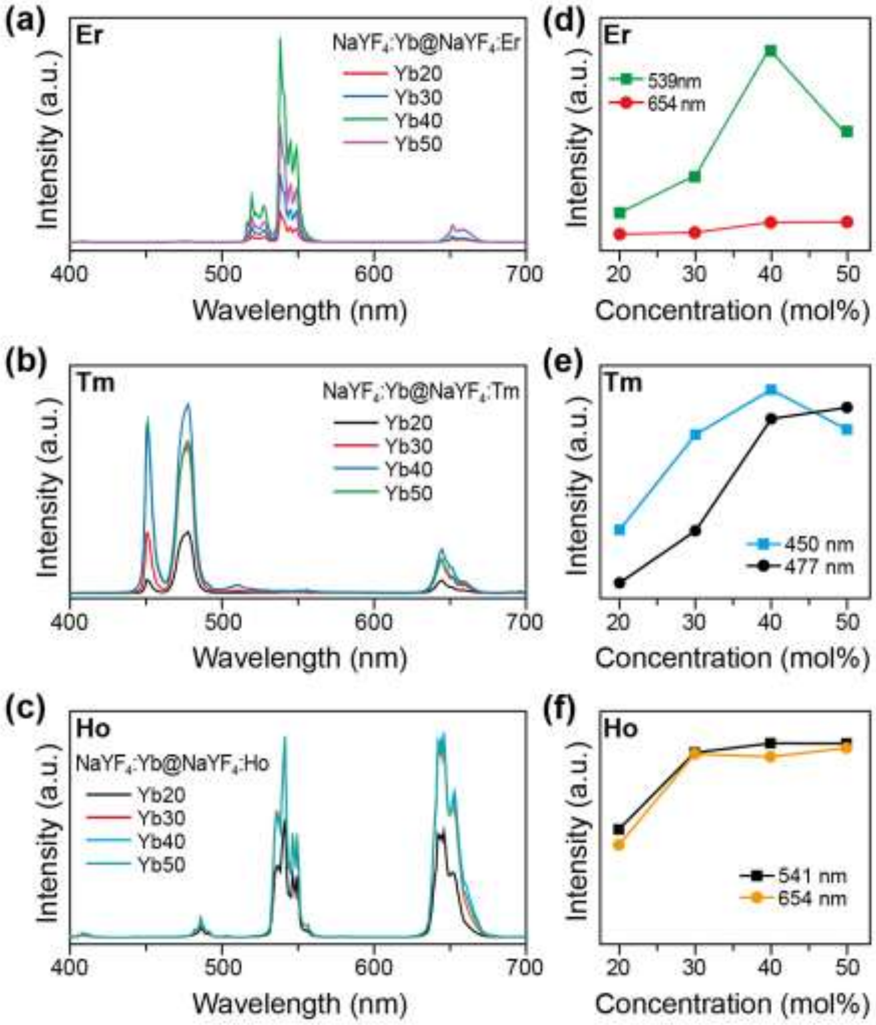

Figure S2. Optimization of photon upconversion from the $NaYF_4$:Yb@$NaYF_4$:A(A=Er, Tm, Ho) core-shell nanoparticles through tuning the concentration of Yb^{3+} sensitizer in the core layer. (a-c) Upconversion emission spectra of $NaYF_4$:Yb(20-50 mol%)@$NaYF_4$:A (A=Er, 2 mol%; Tm, 0.5 mol%; Ho, 2 mol%) core-shell samples with different Yb^{3+} dopant concentration under 980 nm excitation. (d-f) Upconversion luminescence intensity as a function of the Yb^{3+} concentration in the core layers for the core-shell samples in (a-c).

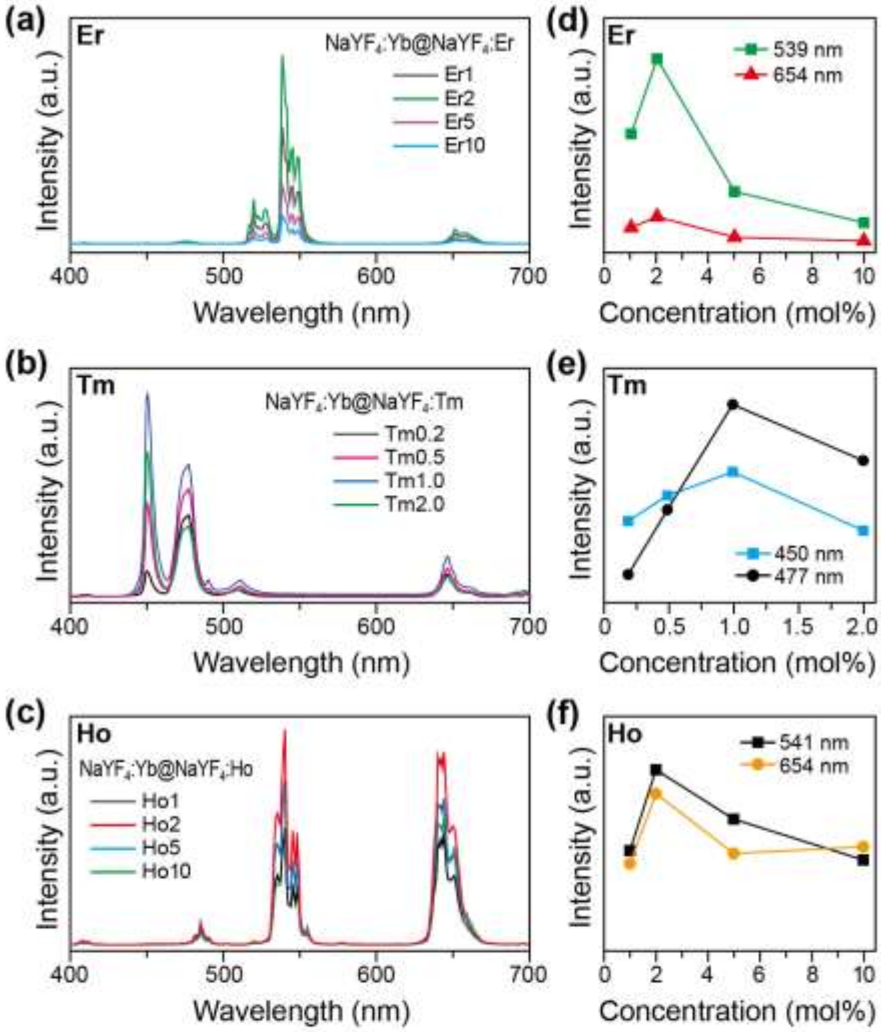

Figure S3. Optimization of photon upconversion from the $NaYF_4$:Yb@$NaYF_4$:A(A=Er, Tm, Ho) core-shell nanoparticles through tuning the concentration of A^{3+} activator in the shell layer. (a-c) Upconversion emission spectra of $NaYF_4$:Yb(40 mol%)@$NaYF_4$:A (A=Er,1-10 mol%; Tm, 0.2-2.0 mol%; Ho, 1-10 mol%) core-shell samples with different A^{3+} dopant concentration under 980 nm excitation. (d-f) Upconversion luminescence intensity as a function of the A^{3+} concentration in the core layers for the core-shell samples in (a-c).

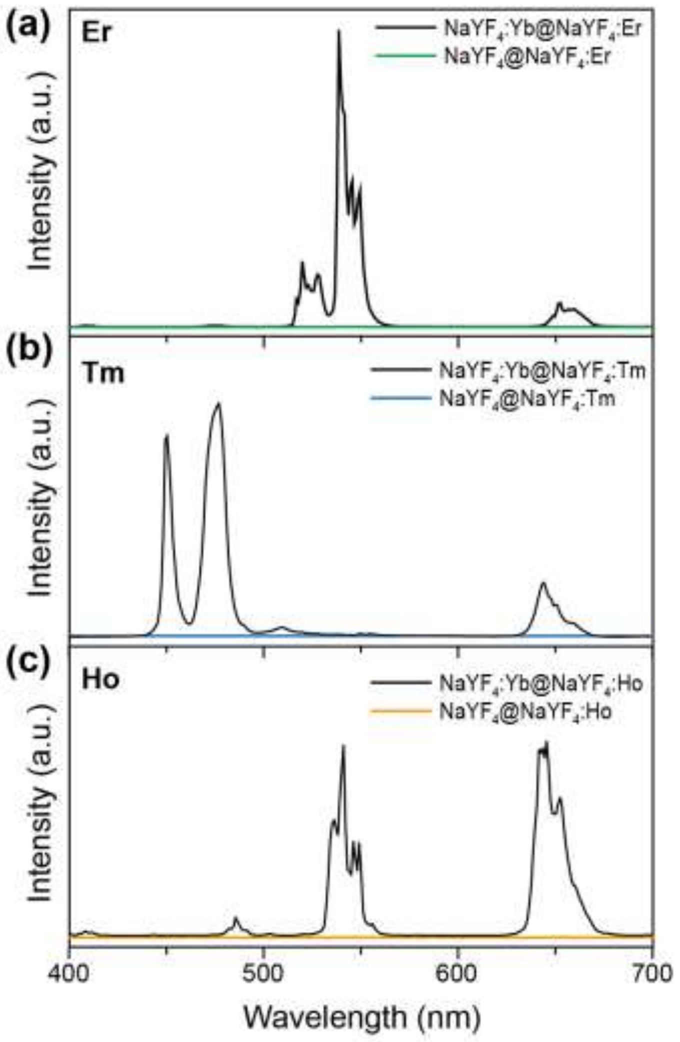

Figure S4. A comparative study of upconversion emission properties in core-shell nanoparticle samples doping with and without Yb^{3+} in the core layer. (a) Upconversion emission spectra of $NaYF_4$:Yb(40 mol%)@$NaYF_4$:Er(2 mol%) and $NaYF_4$@$NaYF_4$:Er(2 mol%) core-shell samples under 980 nm excitation, showing an extremely low contribution of direct excitation of Er^{3+} in the shell layer by the 980 nm excitaiton compared to the IET-mediated upconversion process. (b,c) Upconversion emission spectra of $NaYF_4$:Yb(40 mol%)@$NaYF_4$:A (A=Tm, 0.5 mol%; Ho, 2 mol%) and $NaYF_4$@$NaYF_4$:A (A=Tm, 1 mol%; Ho, 2 mol%) core-shell samples under 980 nm excitation, indicating a nonresponsive feature on the 980 nm lasing source for the core-shell samples doping without Yb^{3+} in the core layer.

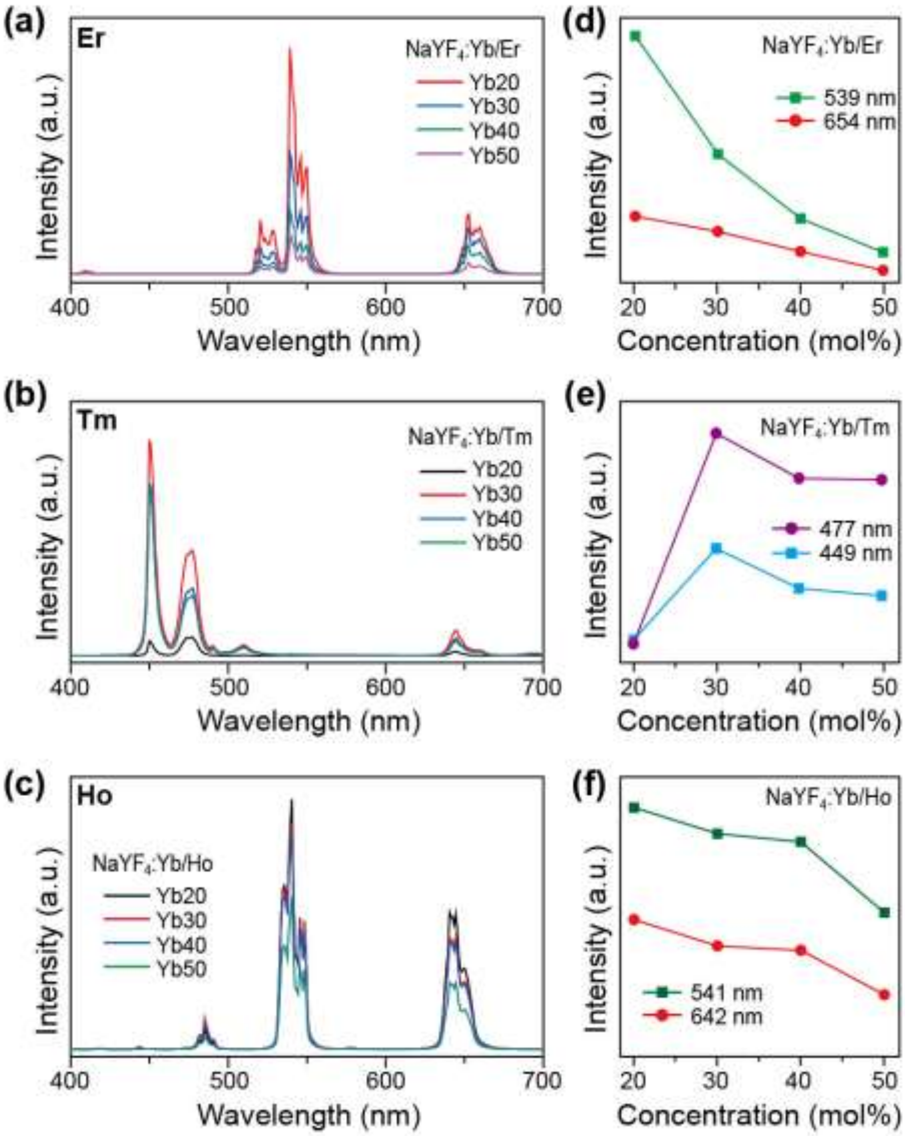

Figure S5. (a-c) Upconversion spectra obtained from (a) $NaYF_4$:Yb/Er (Yb, 20-50 mol%; Er, 2 mol%), (b) $NaYF_4$:Yb/Tm (Yb, 20-50 mol%; Tm, 0.5 mol%) and (c) $NaYF_4$:Yb/Ho (Yb, 20-50 mol%; Ho, 2 mol%) core nanoparticles under 980 nm excitation. (d-f) Dependence of luminescence intensity on Yb^{3+} concentration for upconversion emissions from (d) Er^{3+}, (e) Tm^{3+}, and (f) Ho^{3+}, respectively.

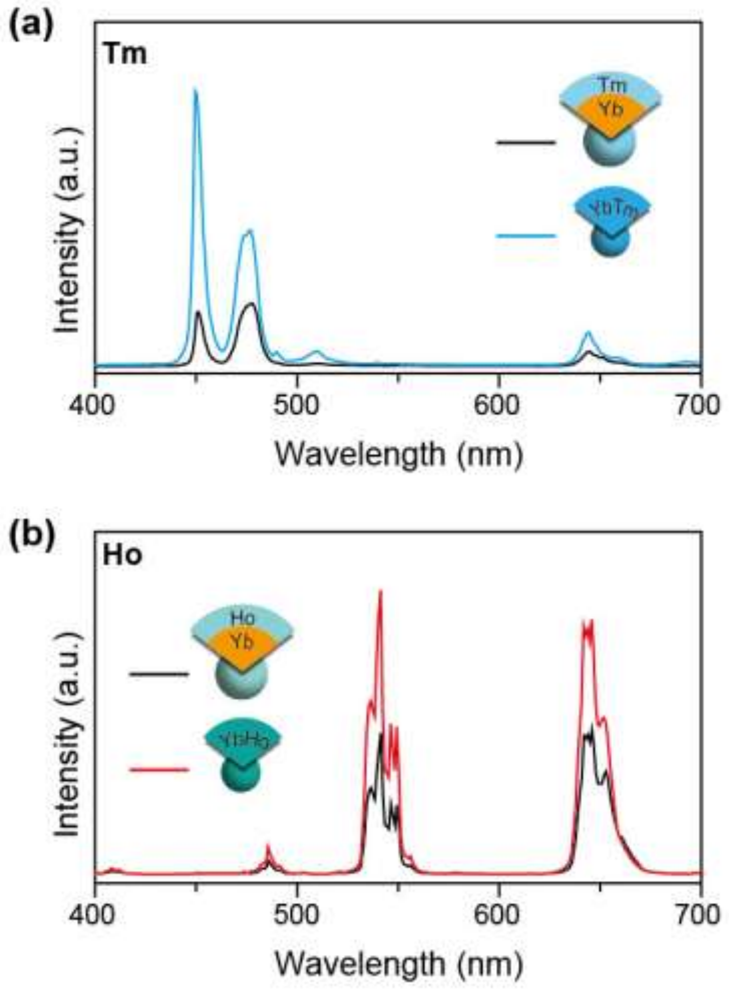

Figure S6. A comparison of upconversion spectra obtained from (a) $NaYF_4$:Yb/Tm(30/0.5 mol%) and $NaYF_4$:Yb(40 mol%)@$NaYF_4$:Tm(0.5 mol%) core-shell samples, and (b) $NaYF_4$:Yb/Ho(20/2 mol%) and $NaYF_4$:Yb(40 mol%)@$NaYF_4$:Ho(2 mol%) core-shell samples upon a 980-nm pulsed laser under identical measurement condition, showing a decline in luminescence intensity compared to the conventionally Yb-A codoped samples.

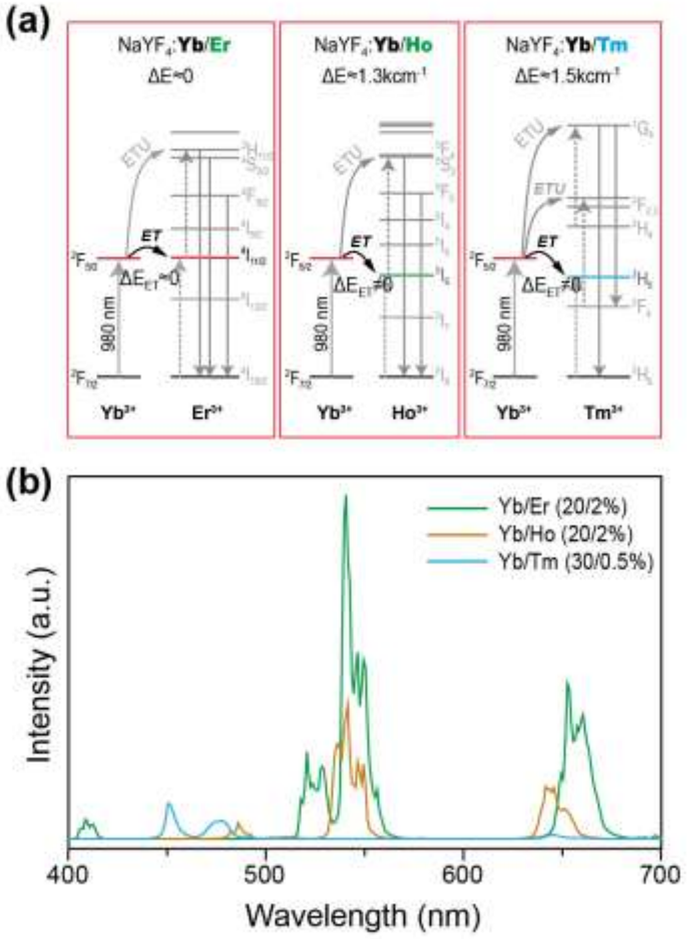

Figure S7. (a) Schematic of energy mismatching for the Yb-Er, Yb-Ho, and Yb-Tm couples with photon upconversion configuration, showing an energy mismatching relationship of $[Yb(^4F_{5/2})\text{-}Er(^4I_{11/2})] < [Yb(^4F_{5/2})\text{-}Ho(^5I_6)] < [Yb(^4F_{5/2})\text{-}Tm(^3H_5)]$. (b) A comparative study of the upconversion emission from $NaYF_4$:Yb/Er(20/2 mol%), $NaYF_4$:Yb/Ho(20/2 mol%), and $NaYF_4$:Yb/Tm(30/0.5 mol%) nanoparticles under 980 nm excitation at identical pump power density (0.8 W/cm^2).

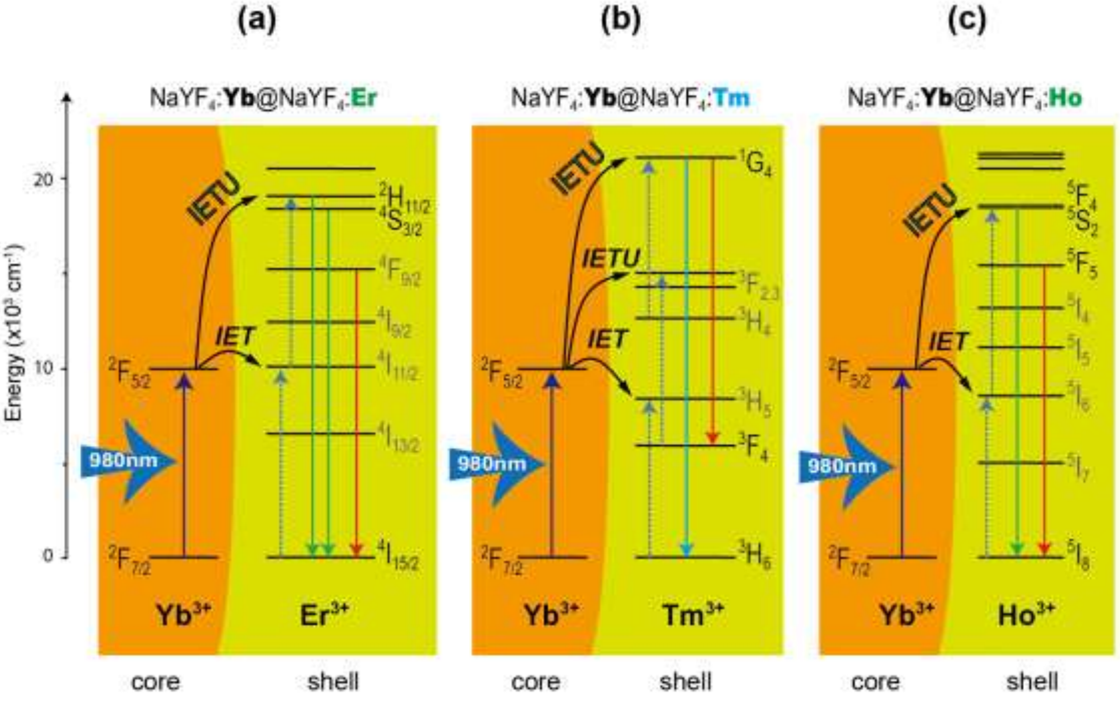

Figure S8. (a-c) Proposed mechanism of photon upconversion through interfacial energy transfer (IET) in a core-shell structure design. The infrared irradiation energy is initially absorbed by Yb^{3+} sensitizer through a ground-state absorption (GSA) process, and then transferred to the lanthanide activators A (A = Er^{3+}, Tm^{3+} and Ho^{3+}) followed by a Yb^{3+}-mediated IET process across the core-shell interfacial area (here Yb^{3+} ions act as both energy donor and sensitizer), resulting in visible upconversion emissions of these lanthanide emitters. Note that the typical optical transitions for visible emissions are indicated: (i) Er^{3+}: $^2H_{11/2}$,$^4S_{3/2}$ $\rightarrow$ $^4I_{15/2}$ (green emission) and Er^{3+}: $^4F_{9/2}$ $\rightarrow$ $^4I_{15/2}$ (red emission); (ii) Tm^{3+}: $^1G_4$ $\rightarrow$ $^3H_6$ (blue emission) and Tm^{3+}: $^1G_4$ $\rightarrow$ $^3F_4$ (red emission); (iii) Ho^{3+}: $^5F_4$,$^5S_2$ $\rightarrow$ $^5I_8$ (green emission) and Ho^{3+}: $^5F_5$ $\rightarrow$ $^5I_8$ (red emission).

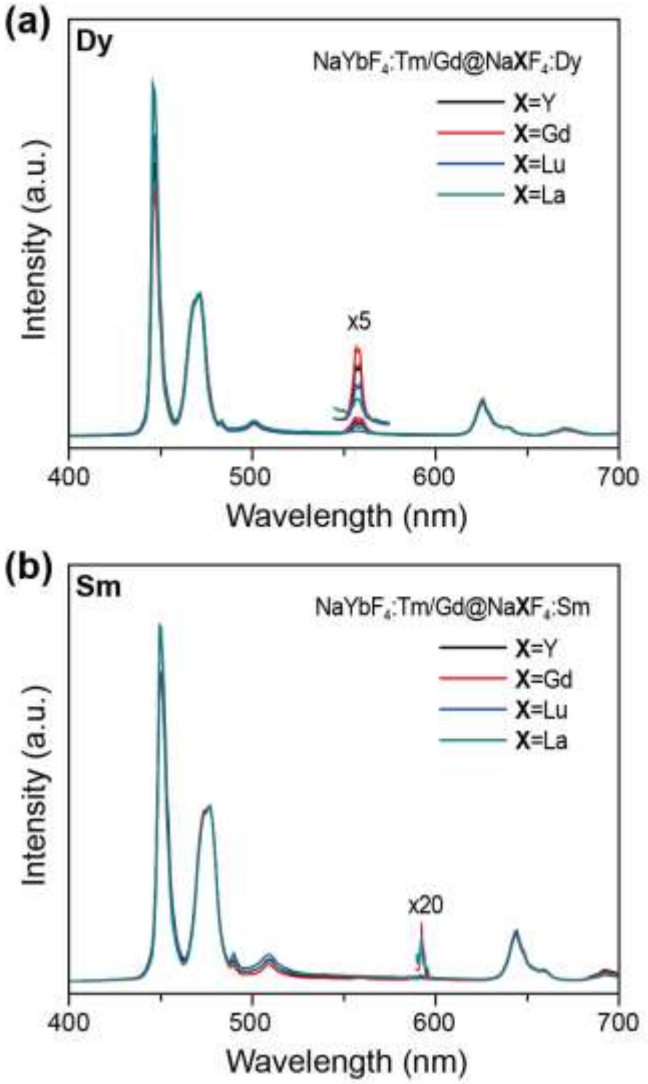

Figure S9. Upconversion emission spectra obtained from (a) $NaYbF_4$:Tm/Gd@$NaXF_4$:Dy (X=Y, Gd, Lu, La) and (b) $NaYbF_4$:Tm/Gd@$NaXF_4$:Sm (X=Y, Gd, Lu, La) core-shell nanoparticles under 980 nm excitation. Note that the spectra are enlarged for a clear comparison. Note that the recorded weak emissions of Dy^{3+} emission is from $^4F_{9/2} \rightarrow {}^6H_{13/2}$ transition peaking at 570 nm, and that of Sm^{3+} is from $^4G_{5/2} \rightarrow {}^6H_{7/2}$ transition peaking at 594 nm.

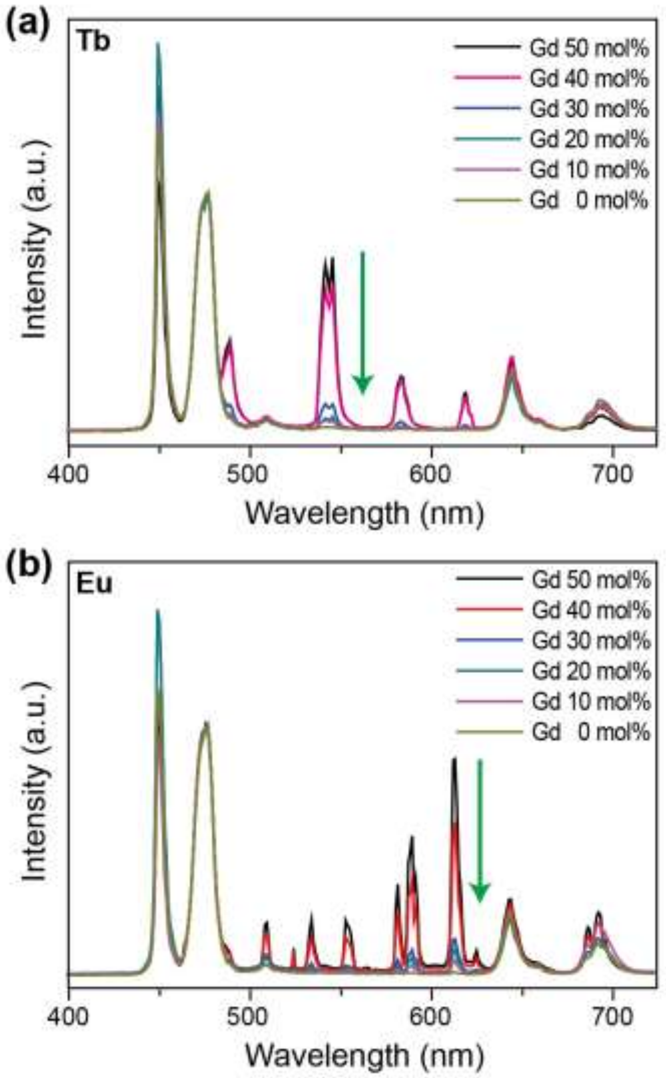

Figure S10. (a,b) Upconversion emission spectra of $NaYbF_4$:Tm/Gd/Y(1/50-*x*/*x* mol%)@$NaYbF_4$:Tm/Gd(1/50 mol%)@$NaYF_4$:A (A = Tb, Eu; 5 mol%) tri-layer samples under 980 nm excitation. Note that the content of Gd^{3+} in the core layer is tuned by the use of inactive Y which increases from 0 to 50 mol% in concentration. Note that the recorded emissions of Eu^{3+} are from transitions of $^5D_2 \rightarrow {}^7F_3$ (510 nm), $^5D_1 \rightarrow {}^7F_0$ (525 nm), $^5D_1 \rightarrow {}^7F_2$ (555 nm), $^5D_1 \rightarrow {}^7F_3$ (584 nm), $^5D_0 \rightarrow {}^7F_{0,1}$ (591 nm), $^5D_0 \rightarrow {}^7F_2$ (614 nm) and $^5D_0 \rightarrow {}^7F_4$ (695 nm), while the emissions of Tb^{3+} are from $^5D_4 \rightarrow {}^7F_6$ (nm), $^5D_4 \rightarrow {}^7F_5$ (nm), $^5D_4 \rightarrow {}^7F_4$ (nm) and $^5D_4 \rightarrow {}^7F_3$ (nm).

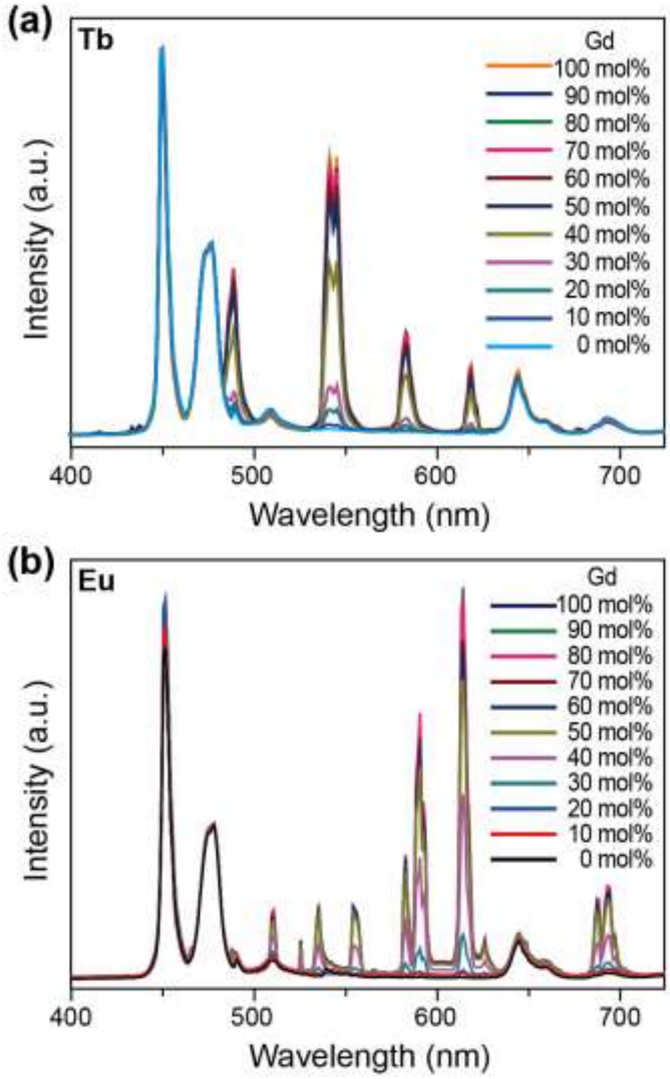

Figure S11. (a,b) Upconversion emission spectra of $NaYbF_4$:Tm/Gd(1/50 mol%)@$NaYF_4$: Gd(0-100 mol%)@$NaYF_4$:A (A = Tb, Eu; 5 mol%) tri-layer nanoparticle samples under 980 nm excitation, showing a rapid rise in emissin intensity as the concentration of Gd^{3+} in the interlayer reaches and above 40 mol%.

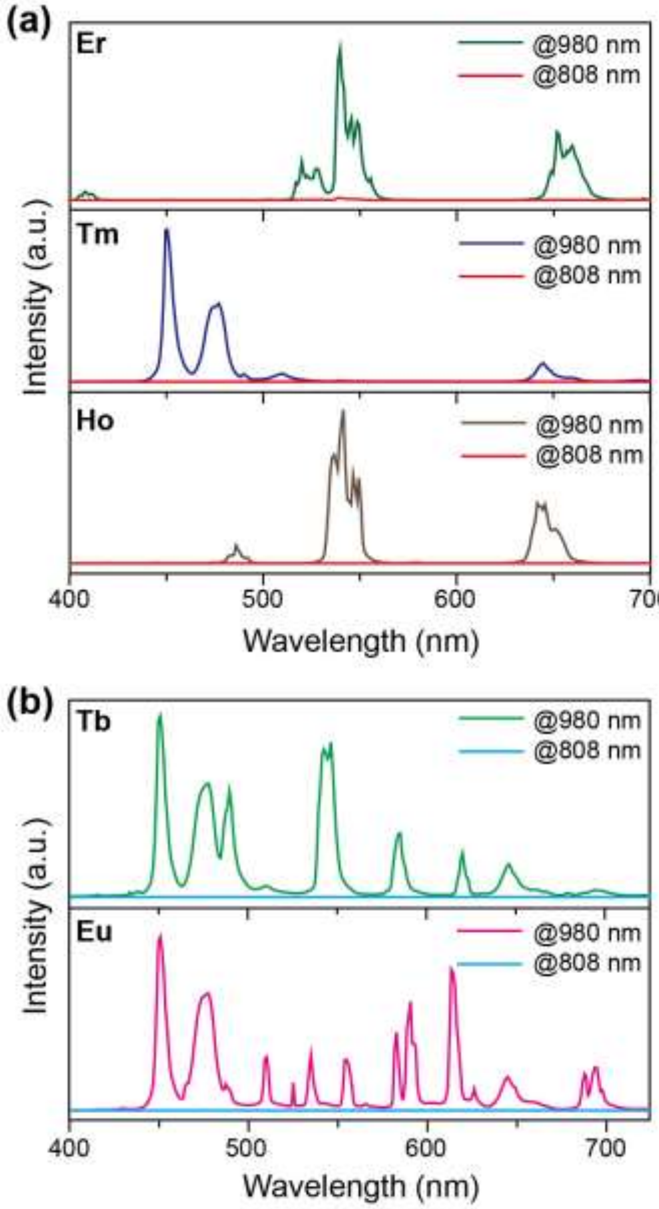

Figure S12. (a) Upconversion emission spectra from $NaYF_4$:Yb/Er(20/2 mol%), $NaYF_4$:Yb/Tm(30/0.5 mol%), and $NaYF_4$:Yb/Ho(20/2 mol%) core samples under 980 and 808 nm excitation. (b) Upconversion emission spectra from $NaYbF_4$:Tm/Gd (1/50 mol%)@$NaYF_4$:A(A=Tb,Eu; 5 mol%) core-shell samples under 980 and 808 nm excitation.

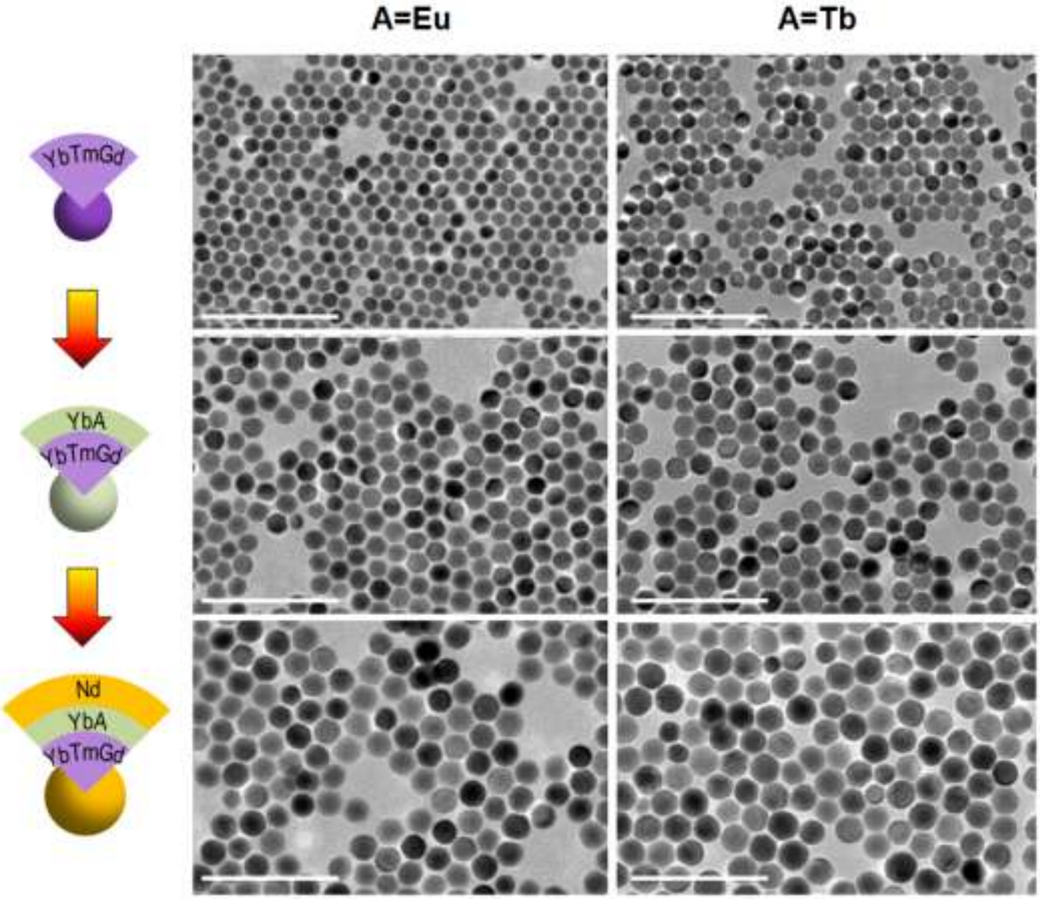

Figure S13. TEM images of $NaYbF_4$:Tm/Gd(1/50 mol%) core, $NaYbF_4$:Tm/Gd(1/50 mol%)@$NaYF_4$:Yb/A(A=Eu, Tb) core-shell, and $NaYbF_4$:Tm/Gd(1/50 mol%)@$NaYF_4$:Yb/A(A=Eu, Tb)@$NaYbF_4$:Nd core-shell-shell nanoparticles. Note that the left panels are Eu-coupled system and the right panels are Tb-coupled system, respectively. Scale bars are 200 nm.

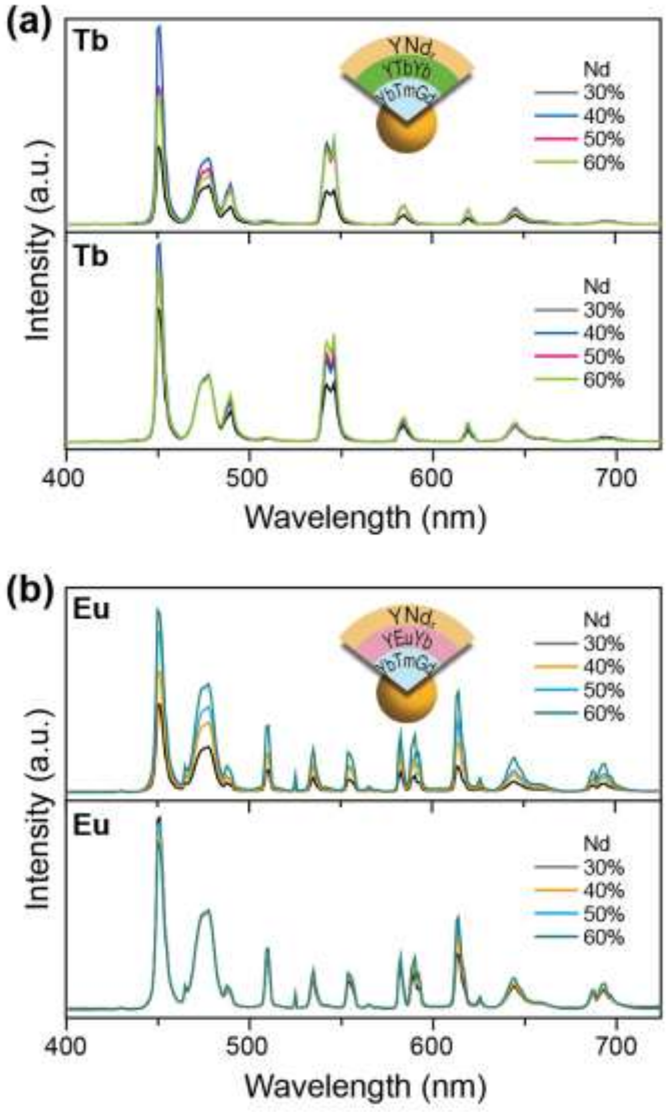

Figure S14. Photon upconversion from the $NaYbF_4$:Tm/Gd(1/50 mol%)@$NaYF_4$:Yb/A(A= Eu,Tb)@$NaYbF_4$:Nd core-shell-shell samples through tuning the doping concentration of Nd^{3+} sensitizer in the outermost shell layer. (a,b) Upconversion emission spectra of $NaYbF_4$:Tm/Gd(1/50 mol%)@$NaYF_4$:Yb/A(Yb, 40 mol%; A=Eu,Tb, 5 mol%)@$NaYbF_4$:Nd (30-60 mol%) core-shell samples with different Nd^{3+} dopant concentration under 808 nm excitation. Note that the spectra plotted in top panels are the originally recorded data and that in the bottom panels are the ones normalized to the Tm^{3+} emission at 477 nm.

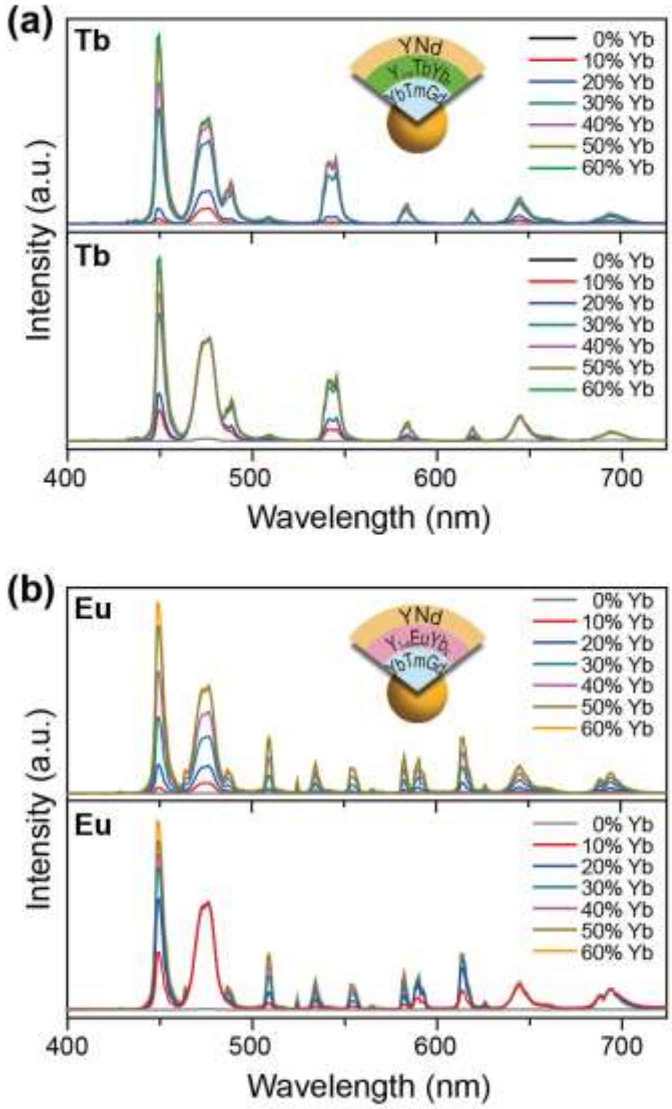

Figure S15. Photon upconversion from the $NaYbF_4$:Tm/Gd(1/50 mol%)@$NaYF_4$:Yb/A(A= Eu,Tb)@$NaYbF_4$:Nd core-shell-shell samples through tuning the dopant concentration of Yb^{3+} in the inter-layer. (a,b) Upconversion emission spectra of $NaYbF_4$:Tm/Gd(1/50 mol%)@$NaYF_4$:Yb/A(Yb, 0-60 mol%; A=Eu,Tb, 5 mol%)@$NaYbF_4$:Nd(50 mol%) core-shell samples with different Yb^{3+} dopant concentration under 808 nm excitation. Note that the spectra plotted in top panels are the originally recorded data and that in the bottom panels are the ones normalized to the Tm^{3+} emission at 477 nm.

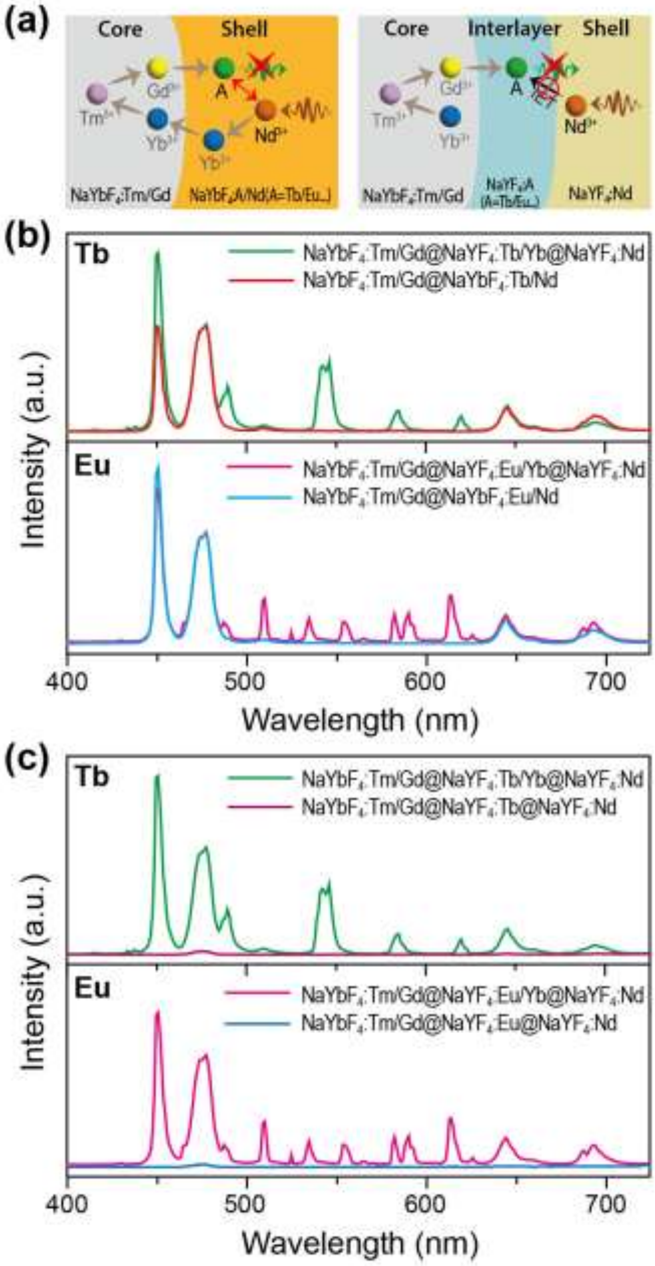

Figure S16. (a) Schematic of interactions between sensitizer Nd^{3+} and activator A (A=Tb^{3+}, Eu^{3+}). Left panel, interaction between Nd^{3+} and A results in luminescence quenching; Right panel, IET from Nd^{3+} in the outermost layer to the A in the interlayer does not lead to the emission of A. (b) Upconversion emission spectra from $NaYbF_4$:Tm/Gd(1/50 mol%)@$NaYF_4$:Yb/A(A=Eu, Tb)@$NaYbF_4$:Nd(50 mol%) core-shell-shell and $NaYbF_4$:Tm/Gd(1/50 mol%)@$NaYbF_4$:A/Nd(A=Eu,Tb, 5 mol%; Nd, 50 mol%) core-shell samples under 808 nm excitation. (c) Upconversion emission spectra from $NaYbF_4$:Tm/Gd(1/50 mol%)@$NaYF_4$:Yb/A(A=Eu, Tb)@$NaYbF_4$:Nd(50 mol%) and $NaYbF_4$:Tm/Gd(1/50 mol%)@$NaYF_4$:A(A=Eu, Tb)@$NaYbF_4$:Nd(50 mol%) core-shell-shell nanoparticles under 808 nm excitations.

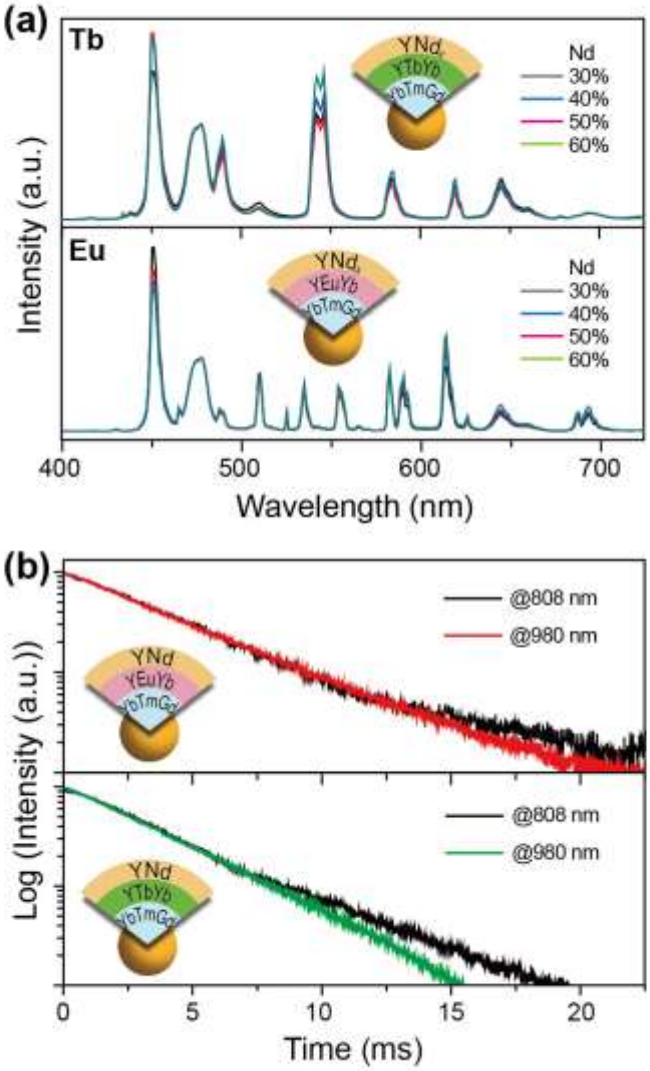

Figure S17. (a) Upconversion emission spectra obtained from (top panel) $NaYbF_4$:Tm/Gd(1/50 mol%)@$NaYF_4$:Yb/Tb(40/5 mol%)@$NaYF_4$:Nd(30-60 mol%) and (bottom panel) $NaYbF_4$:Tm/Gd(1/50 mol%)@$NaYF_4$:Yb/Eu(40/5 mol%)@$NaYF_4$:Nd(30-60 mol%) trilayer nanoparticles under 980 nm excitation. (b) Upconversion decay curves obtained from (top panel) Tb^{3+} emission at 545 nm ($^5D_4 \rightarrow {}^7F_5$ transition) from the $NaYbF_4$:Tm/Gd(1/50 mol%)@$NaYF_4$:Yb/Tb(40/5 mol%)@$NaYbF_4$:Nd(50 mol%), and(bottom panel) from Eu^{3+} emission at 614 nm ($^5D_0 \rightarrow {}^7F_2$ transition) from the $NaYbF_4$:Tm/Gd(1/50 mol%)@$NaYF_4$:Yb/Eu(40/5 mol%)@$NaYbF_4$:Nd(50 mol%) tri-layer samples under pulsed 980 and 808 nm excitation.

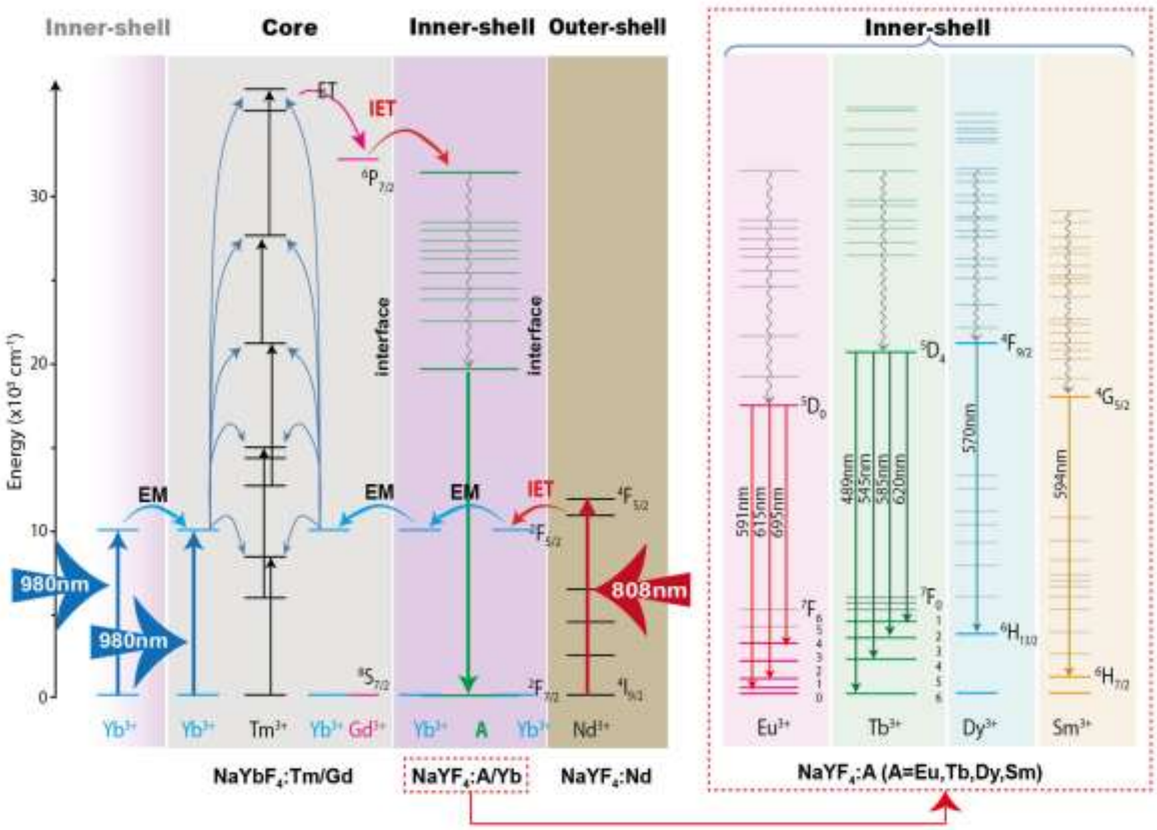

Figure S18. Proposed mechanism of photon upconversion through interfacial energy transfer (IET) in a core-shell-shell structure design under 808 nm excitation and 980/808 nm dual wavelength excitation. The infrared irradiation energy at 808 nm is initially absorbed by Nd^{3+} sensitizer through a ground-state absorption (GSA) process, and then transferred to the Yb^{3+}-Tm^{3+}-Gd^{3+} coupled sensitizer system followed by multiple IET and EM processes across the interfacial areas and inner-shell layers before activating the lanthanide activators A (A = Tb^{3+}, Eu^{3+} and Dy^{3+}) in the inner-shell layer, resulting in visible upconversion emissions of these lanthanide emitters. Note that the typical optical transitions for visible emissions are indicated.

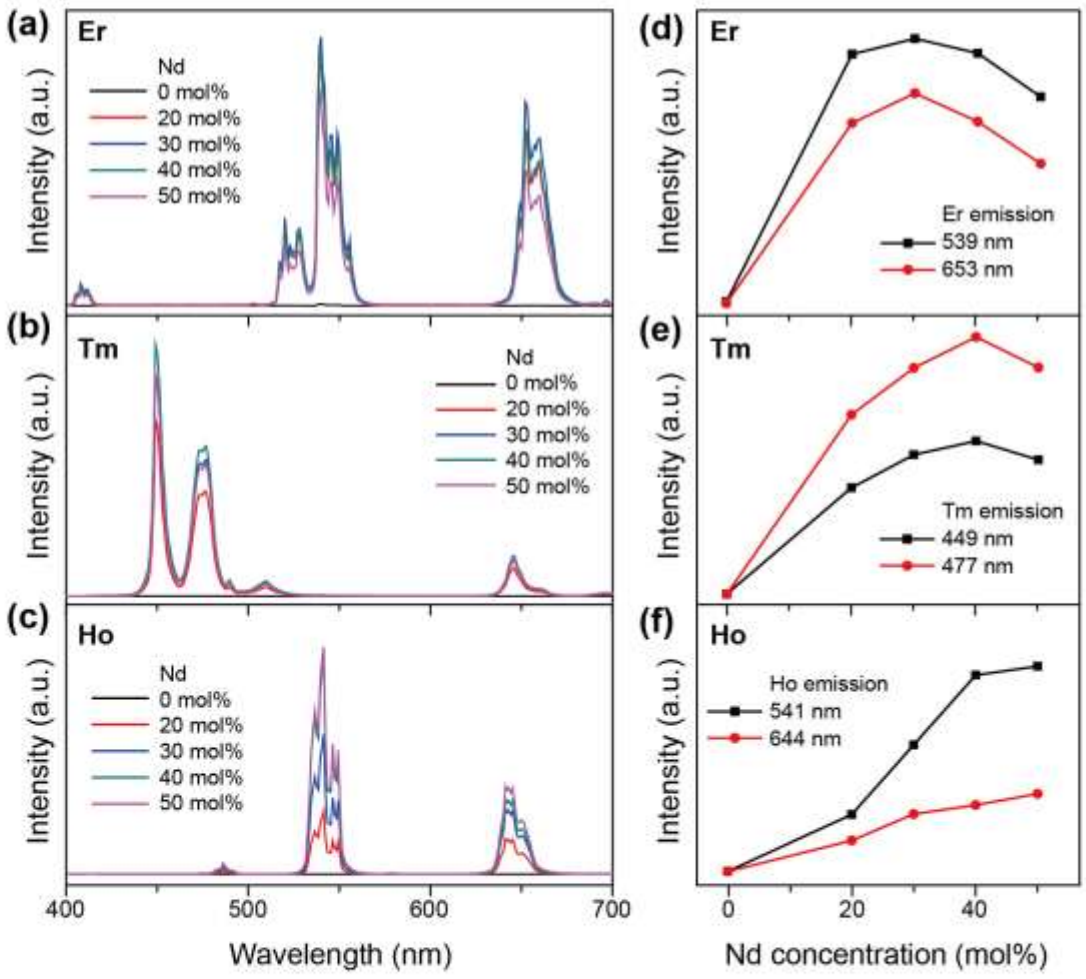

Figure S19. (a-c) Upconversion emission spectra of $NaYF_4$:Yb/A(A=Er,Tm,Ho; 20/2, 30/0.5, 20/2 mol%)@$NaYF_4$:Nd(0-60 mol%) core-shell samples with different Nd^{3+} dopant concentration under 980 nm excitation. (d-f) Upconversion luminescence intensity as a function of Nd^{3+} concentration in the shell layers for the core-shell samples in (a-c).

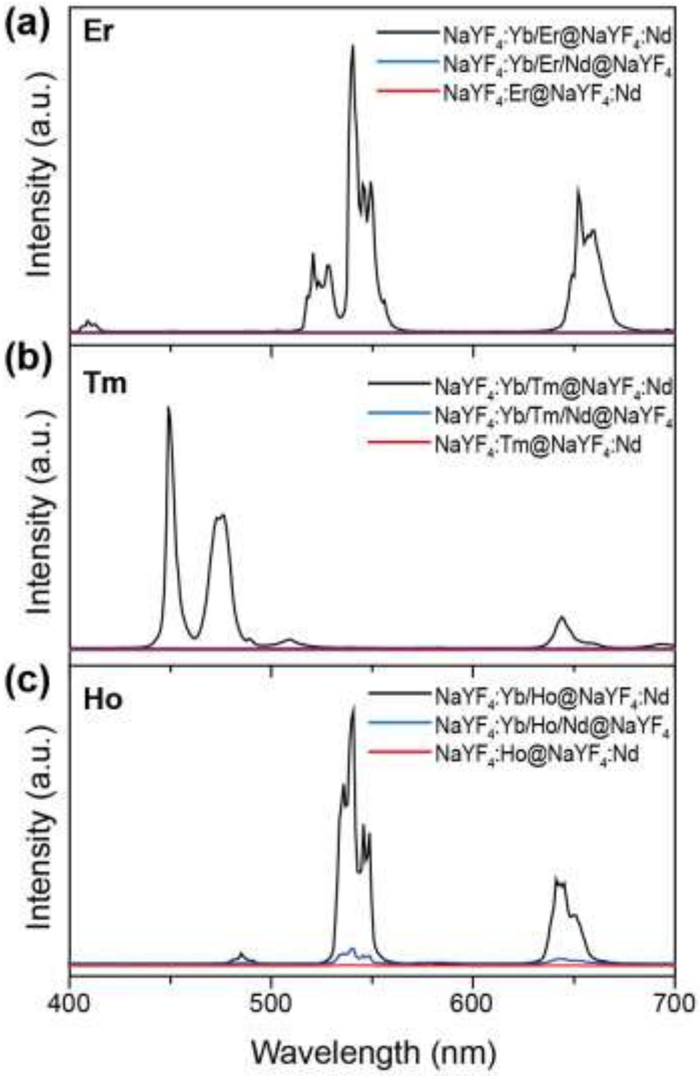

Figure S20. Comparative upconversion emission spectra from (a) $NaYF_4$:Yb/Er(20/2 mol%)@$NaYF_4$:Nd(30 mol%), $NaYF_4$:Yb/Er/Nd(20/2/30 mol%)@$NaYF_4$ and $NaYF_4$: Er(2 mol%)@$NaYF_4$:Nd(30 mol%) core-shell samples, (b) $NaYF_4$:Yb/Tm(30/0.5 mol%)@$NaYF_4$:Nd(50 mol%), $NaYF_4$:Yb/Tm/Nd(30/0.5/40 mol%)@$NaYF_4$ and $NaYF_4$:Tm(0.5 mol%)@$NaYF_4$:Nd(40 mol%) core-shell samples, and (c) $NaYF_4$:Yb/Ho(20/2 mol%)@$NaYF_4$:Nd(40 mol%), $NaYF_4$:Yb/Ho/Nd(20/2/40 mol%)@$NaYF_4$ and $NaYF_4$:Ho(2 mol%)@$NaYF_4$:Nd(40 mol%) core-shell samples under 808 nm excitation.

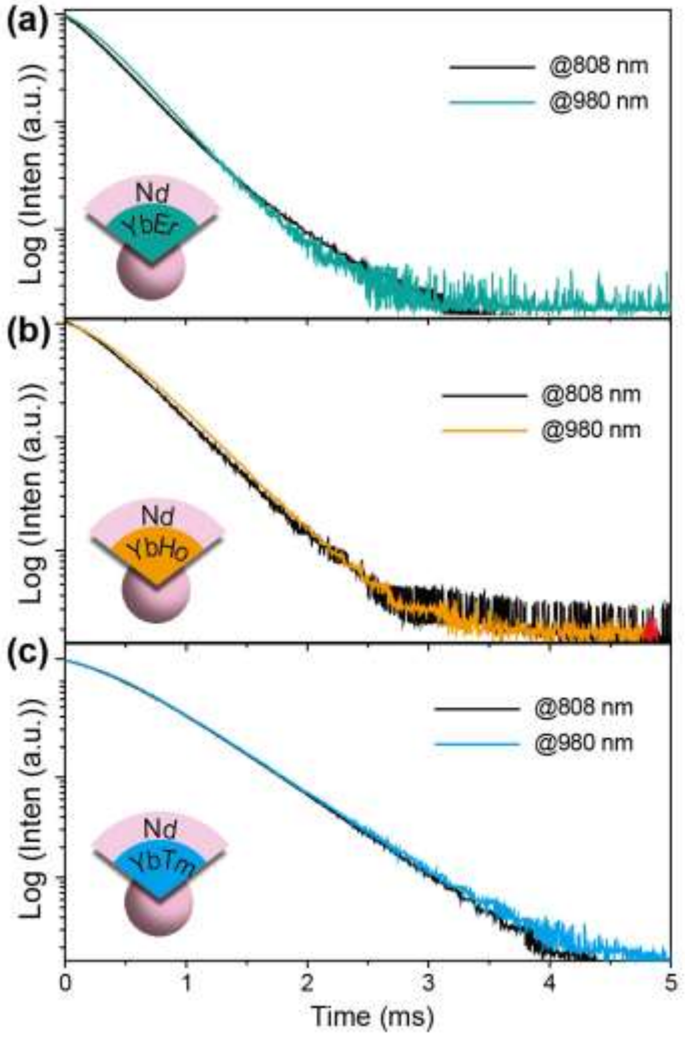

Figure S21. Upconversion decay curves obtained from (a) Er^{3+} emission at 539 nm ($^4S_{3/2} \rightarrow {}^4I_{15/2}$ transition) from the $NaYF_4$:Yb/Er(20/2 mol%)@$NaYF_4$:Nd(40 mol%), (b) Ho^{3+} emission at 541 nm ($^5F_4, {}^5S_2 \rightarrow {}^5I_8$ transition) from the $NaYF_4$:Yb/Ho(20/2 mol%)@$NaYF_4$:Nd(40 mol%), and (c) Tm^{3+} emission at 477 nm ($^1G_4 \rightarrow {}^3H_6$ transition) from the $NaYF_4$:Yb/Tm(20/0.5 mol%)@$NaYF_4$:Nd(40 mol%) core-shell samples under pulsed 980 and 808 nm excitation.

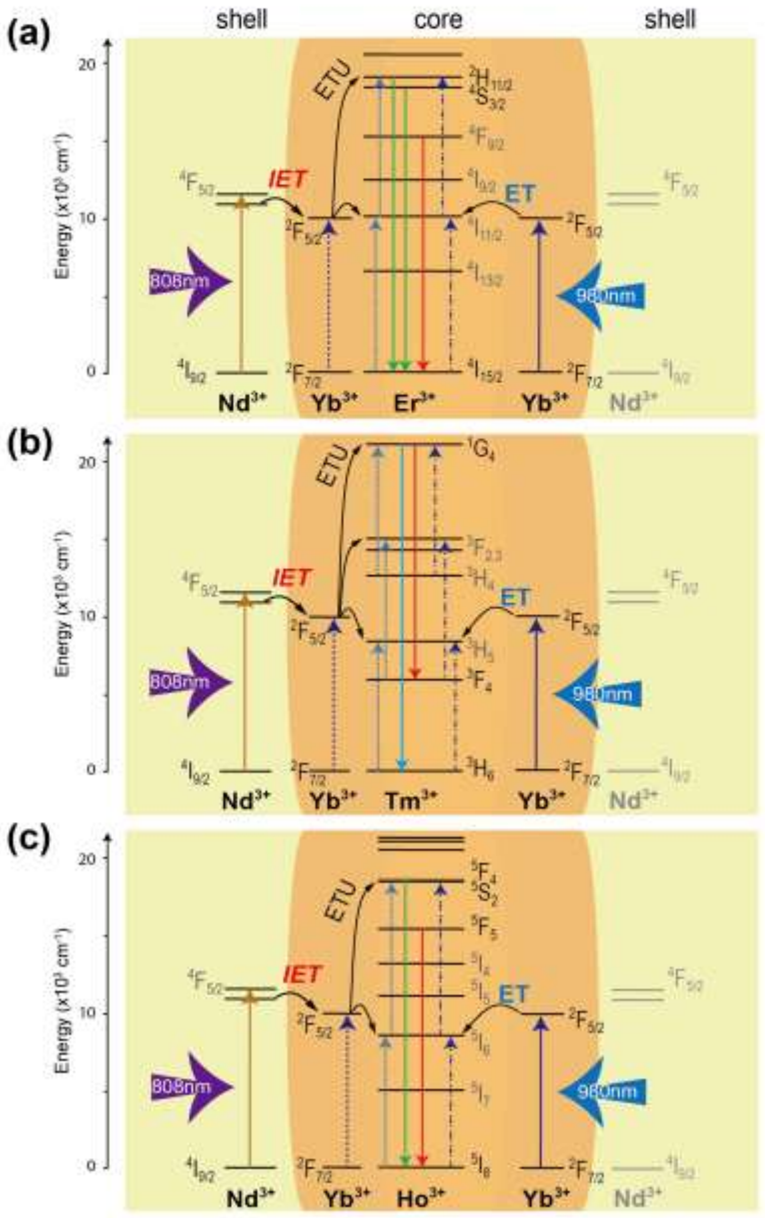

Figure S22. (a-c) Proposed mechanism of 808/980 nm dual-wavelength excitation for photon upconversion at through Nd^{3+}-Yb^{3+} coupled system in a core-shell structure. The infrared irradiation energy at 808 nm is initially absorbed by Nd^{3+} sensitizer in shell layer through a ground-state absorption (GSA) process, and then transferred to the Yb^{3+} sensitizer in core layer followed by an IET process across the core-shell interfacial area before activating the lanthanide activators A (A = Er^{3+}, Tm^{3+} and Ho^{3+}), while the energy at 980 nm irradiation is directly absorbed by the Yb^{3+} in the core layer, resulting in visible upconversion emissions of the lanthanide emitters. Note that the typical optical transitions for visible emission bands from Er^{3+}, Tm^{3+} and Ho^{3+} are indicated.

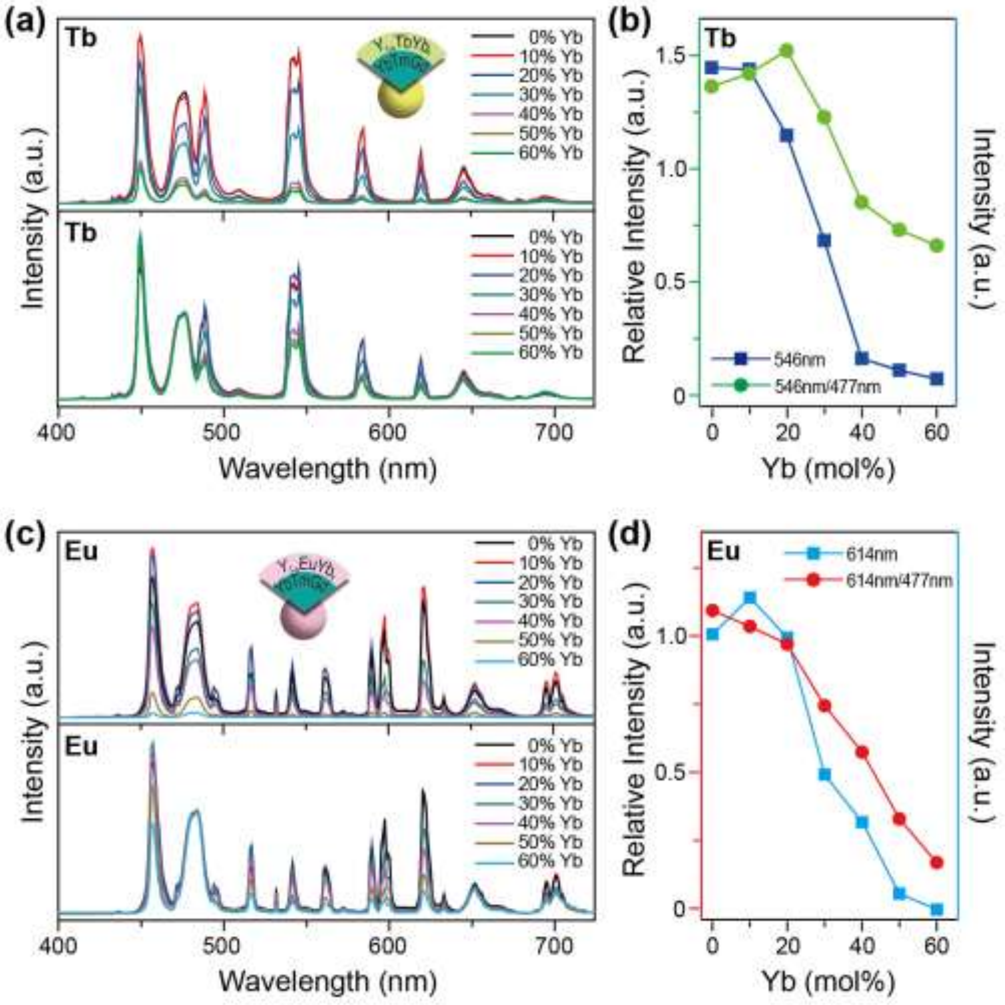

Figure S23. (a,c) Upconversion emission spectra of $NaYbF_4$:Tm/Gd(1/50 mol%)@$NaYF_4$:Yb/A(Yb, 0-60 mol%; A=Eu,Tb, 5 mol%) core-shell samples with different Yb^{3+} dopant concentration under 980 nm excitation. (b,d) Upconversion luminescence intensity as a function of the Yb^{3+} concentration in the shell layer for the core-shell samples in (a,c), showing a rapid decline in emission intensity for the tri-layer samples with Yb^{3+} concentration in the shell layer at high doping levels.

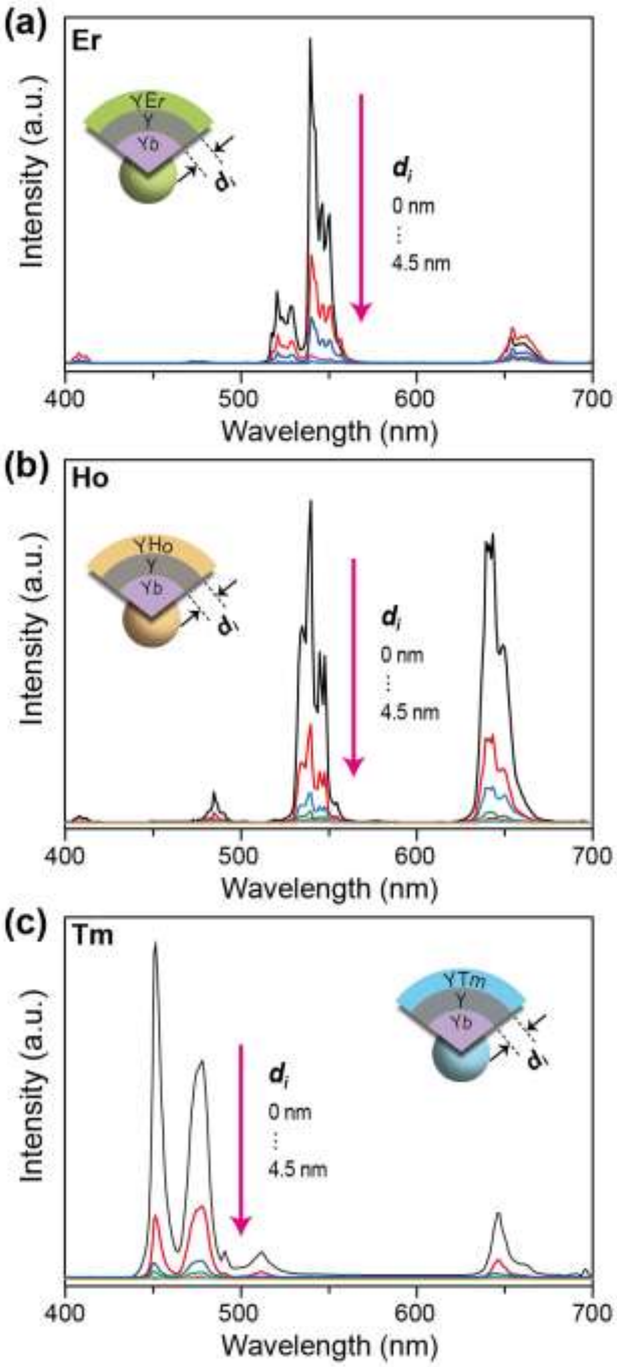

Figure S24. Upconversion emission spectra obtained from $NaYF_4$:Yb@$NaYF_4$@$NaYF_4$:A(A = Er, Tm, Ho) tri-layer nanoparticle samples with different inactive $NaYF_4$ interlayer thickness under 980 nm excitation. (a): $NaYF_4$:Yb(40 mol%)@$NaYF_4$@$NaYF_4$:Er(2 mol%) tri-layer samples; (b): $NaYF_4$:Yb(40 mol%)@$NaYF_4$@$NaYF_4$:Tm(1 mol%) tri-layer samples; (c): $NaYF_4$:Yb(50 mol%)@$NaYF_4$@$NaYF_4$:Ho(2 mol%) tri-layer samples. The inactive $NaYF_4$ interlayer thickness is finely tuned from 0 to 4.5 nm.

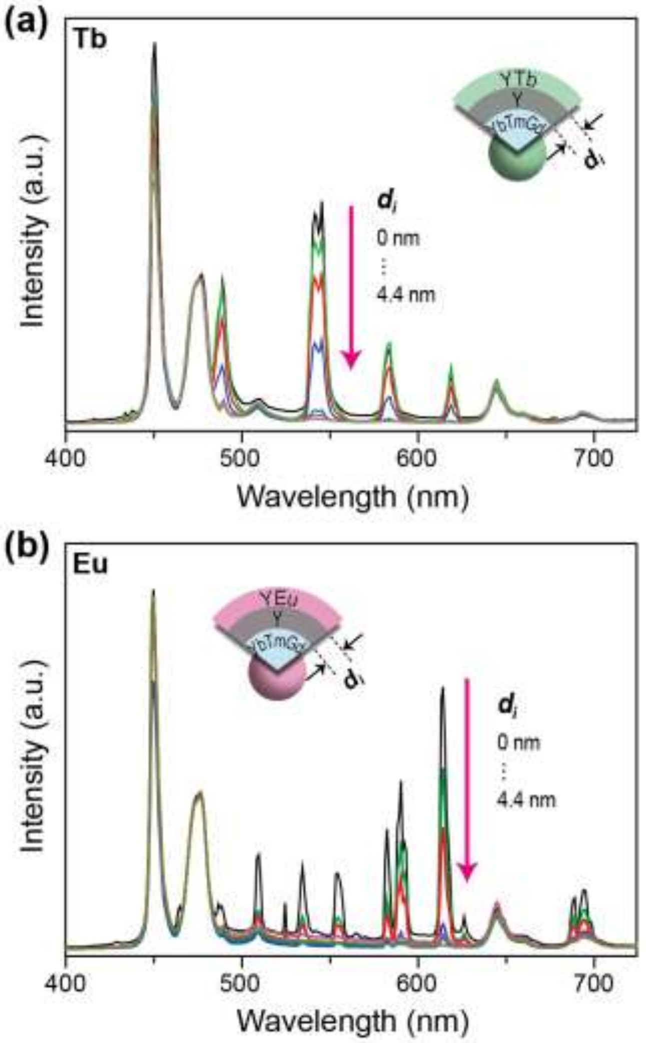

Figure S25. (a,b) Upconversion emission spectra obtained from $NaYbF_4$:Tm/Gd(1/50 mol%)@$NaYF_4$@$NaYF_4$:A(A=Tb,Eu; 5 mol%) tri-layer nanoparticle samples with different inactive $NaYF_4$ interlayer thickness under 980 nm excitation, showing a rapid decline in upconversion emission intensity with increasing the inactive $NaYF_4$ interlayer thickness for both Tb- and Eu-coupled systems.

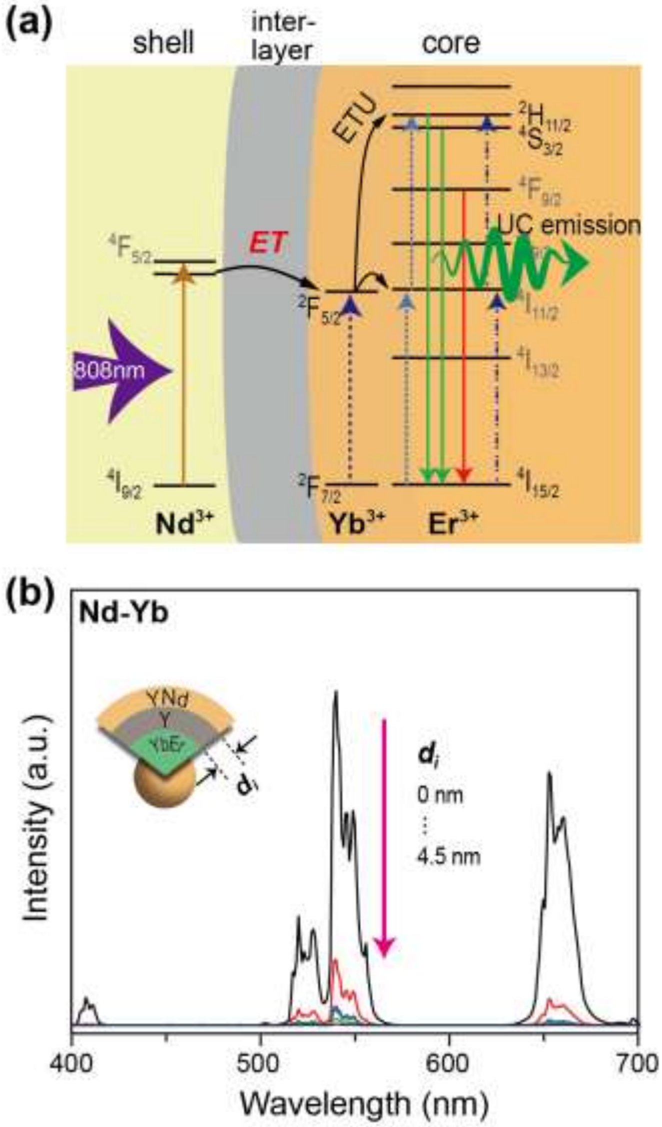

Figure S26. (a) Schematic illustration of controlling energy transfr from Nd^{3+} to Yb^{3+} using a trilayer nanostructure with thickness-tunable interlayer, allowing a detection of the Nd^{3+}-to-Yb^{3+} energy transfer by recording the upconversion emissions from the core layer at 808 nm excitation. (b) Upconversion emission spectra obtained from $NaYF_4$:Yb/Er(20/2 mol%)@$NaYF_4$@$NaYF_4$:Nd(50 mol%) tri-layer nanoparticle samples under 980 nm excitation, showing a rapid decline in upconversion emission intensity with increasing the inactive $NaYF_4$ interlayer thickness.
